# Relationship between stress hyperglycemia ratio and gastrointestinal bleeding and mortality after coronary artery bypass grafting: a multicenter cohort study

**DOI:** 10.3389/fcvm.2026.1873683

**Published:** 2026-07-22

**Authors:** Jiale Dong, Jian Yang, Zhechuan Jin, Yi Jiang, Chengxiang Li, Dongjin Wang, Huachong Ma, Zhili Ji

**Affiliations:** 1 Department of Acute Abdomen Surgery, General Surgery Center, Beijing Chaoyang Hospital Affiliated to Capital Medical University, Beijing, China; 2 Department of Hepatobiliary and Pancreaticosplenic Surgery, General Surgery Center, Beijing Chaoyang Hospital Affiliated to Capital Medical University, Beijing, China; 3 Second Department of Biliary Surgery, Eastern Hepatobiliary Surgery Hospital, Second Military Medical University, Shanghai, China; 4 Cardiac surgery, Nanjing University Medical School Affiliated Nanjing Drum Tower Hospital, Nanjing, China; 5 Beijing Institute of Heart, Lung and Blood Vessel Diseases, Beijing Anzhen Hospital, Capital Medical University, Beijing, China

**Keywords:** adverse outcome, coronary artery bypass grafting, gastrointestinal bleeding, mortality, stress hyperglycemia ratio

## Abstract

**Background:**

Gastrointestinal bleeding after coronary artery bypass grafting (GIBCG) is a serious postoperative complication linked to increased mortality. The stress hyperglycemia ratio (SHR) has been recognized as a reliable predictor of adverse clinical outcomes, particularly in patients with cardiovascular disease. This study aimed to investigate the association between SHR and both GIBCG and in-hospital mortality.

**Methods:**

Patients were recruited from four medical centers and the Medical Information Mart for Intensive Care IV, with a total of 16,440 individuals included in the final analysis. Patients were categorized into four groups based on SHR quartiles. The primary outcomes of interest were the incidence of GIBCG and in-hospital mortality. Restricted cubic splines and logistic regression models were employed to analyze the relationship between SHR and clinical outcomes.

**Results:**

Among the patients who underwent surgery, 1,158 (7.04%) experienced GIBCG, while 214 (1.3%) died during hospitalization. After adjusting for confounding variables, a U-shaped relationship was observed between SHR and both GIBCG occurrence and in-hospital mortality. The lowest incidence of these outcomes was observed within the SHR range of 0.706–0.792, with inflection points identified at 0.71 for GIBCG and 0.733 for in-hospital mortality.

**Conclusion:**

A U-shaped relationship was identified between SHR and both GIBCG and in-hospital mortality. Both low and high SHR levels may help identify patients at increased risk of gastrointestinal bleeding and in-hospital mortality following coronary artery bypass grafting.

## Introduction

Coronary artery disease remains a leading cause of morbidity and mortality worldwide, presenting a significant public health challenge with over 110 million affected individuals globally (1). Coronary artery bypass grafting (CABG) is a standard surgical intervention for severe coronary artery disease, with approximately 370,000 procedures performed annually in the United States (2, 3). Gastrointestinal bleeding (GIB) after coronary artery bypass grafting (GIBCG) is a serious complication. While its incidence is relatively low, ranging from 0.39% to 5.5% (4–9), its clinical impact is profound. Patients experiencing GIBCG face notably higher mortality rates, ranging from 8.8% to 38.0% (4–9). Beyond increased mortality, this complication often leads to prolonged hospital stays and significantly affects patients' quality of life and long-term outcomes.

An elevated stress hyperglycemia ratio (SHR) indicates a temporary rise in blood glucose levels due to physiological or psychological stress (10, 11), often observed in trauma cases and critically ill surgical patients. SHR has been identified as a reliable predictor of adverse outcomes in cardiovascular and cerebrovascular diseases (11–13). Previous research has shown a J-shaped or U-shaped relationship between SHR and both short- and long-term mortality in patients with acute coronary syndrome (11, 13, 14). In patients with severe coronary artery disease undergoing CABG, elevated SHR has been linked to adverse cardiac events (15). The correlation between stress hyperglycaemia and adverse cardiac events in patients with severe coronary heart disease and who undergo CABG has been proven (15). Furthermore, stress hyperglycemia is independently associated with postoperative complications, including cardiac complications, stroke, infections, pneumonia, and acute kidney injury (16–18).

CABG procedures subject patients to considerable physiological stress, given their invasive nature, which includes anesthesia, cardiopulmonary bypass, and surgical trauma (19, 20). These stressors trigger systemic responses, such as the release of stress hormones like cortisol and catecholamines, and a notable increase in blood glucose levels, which may persist postoperatively (21, 22). These reactions, often reflected in an elevated SHR, are part of the body's adaptive mechanisms to manage acute stress. However, they can also increase the risk of complications (21–23). One such complication is GIBCG, with stress-induced mucosal damage or ulcers being a major risk factor (24, 25). Elevated cortisol and catecholamine levels during stress cause vasoconstriction, reducing blood flow to the gastric mucosa. This reduction, combined with increased gastric acid secretion, weakens the mucosal barrier, allowing gastric acid to erode the stomach lining and potentially leading to ulcer formation (26, 27). While these ulcers often affect superficial capillaries, they can also extend deeper into the submucosa, potentially leading to gastrointestinal bleeding or perforation (28). Previous studies suggest a potential association between SHR and GIB. However, it remains unclear whether SHR is associated with GIB and in-hospital mortality following CABG. This study aims to further investigate these associations.

This cohort study has been reported in line with the STROCSS guidelines (29).

## Methods

### Study population and design

Patients were recruited from four medical centers in China. Those who were hospitalized at Beijing Anzhen Hospital between January 2018 and April 2024, and at Nanjing Drum Tower Hospital between January 2018 and May 2023, as well as patients hospitalized at Beijing Luhe Hospital from July 2019 to May 2023, and at Beijing Chaoyang Hospital from May 2022 to April 2024, were included. Additionally, patient data from the Medical Information Mart for Intensive Care IV (MIMIC-IV) was extracted, following the same inclusion and exclusion criteria (30). The study protocol was approved by the Medical Ethics Committee of Beijing Anzhen Hospital Affiliated to Capital Medical University (approval number: KS2023020), and registered with the Chinese Clinical Trial Registry (ChiCTR2400086050).

### Inclusion and exclusion criteria for patients

**Figure 1:**
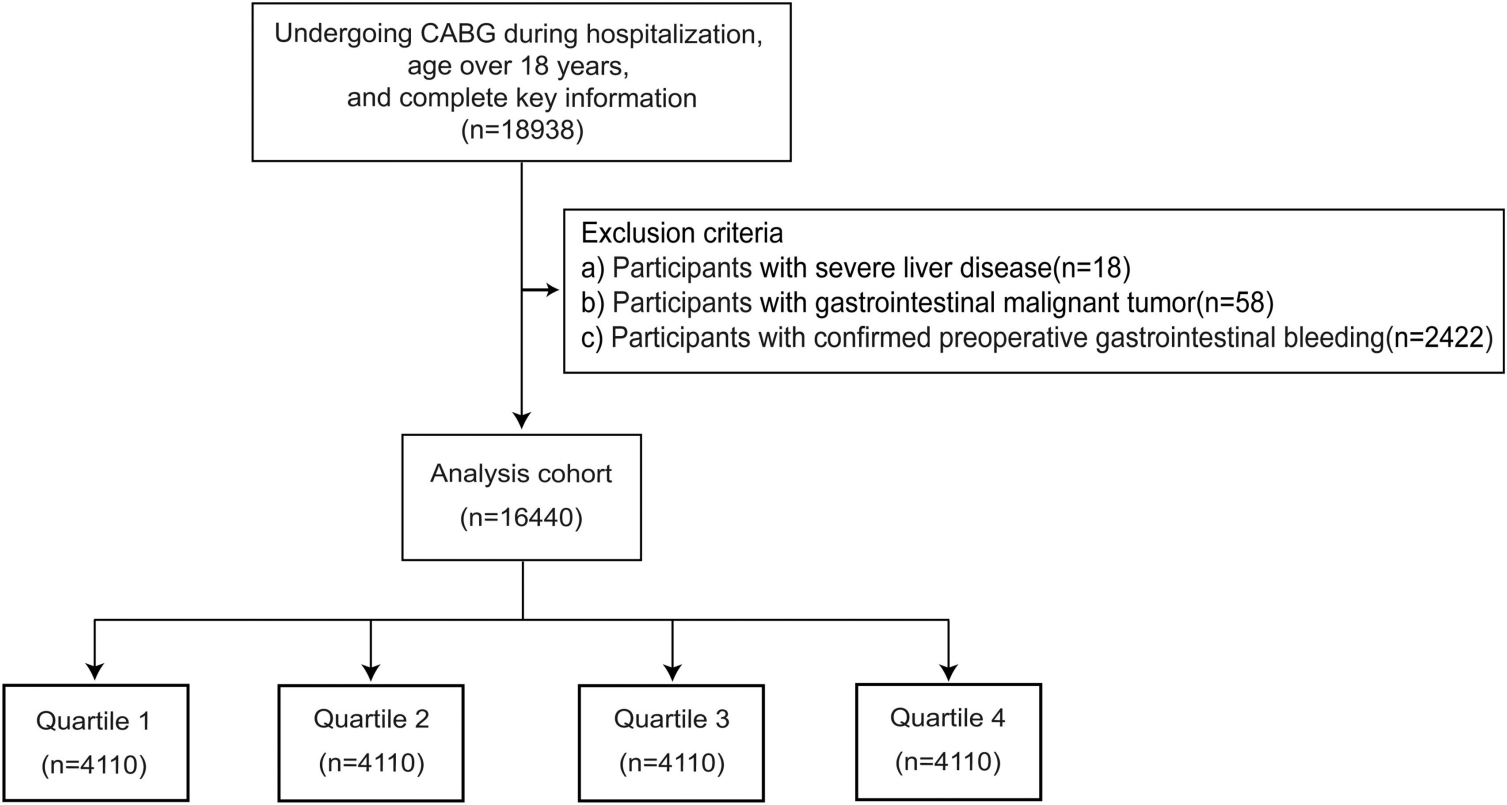
Flow chart.

The inclusion criteria were patients over the age of 18 who underwent CABG during hospitalization. The exclusion criteria were preoperative GIB, severe liver disease, and gastrointestinal malignancy. Patients with severe liver disease or gastrointestinal malignancy were excluded because these conditions may themselves cause gastrointestinal bleeding, making it difficult to determine whether bleeding events were related to CABG. The patient selection process is detailed in Figure 1.

### Definition of outcome

The primary outcome was the development of GIBCG, which was defined by: (1) the presence of hematemesis (including bright red blood or coffee ground emesis), melena, hematochezia, or positive occult blood tests in gastric fluid and stool; and (2) the identification of bleeding sites during endoscopic examination (31–33). The secondary outcome was the rate of in-hospital mortality.

### Data collection and variables

Data collected included patient demographics (age, sex, body mass index), medical history (previous GIB, prior percutaneous coronary intervention), and chronic comorbidities such as chronic anemia, coagulation disorders, hypertension, atrial fibrillation, diabetes mellitus, heart failure, cerebrovascular disease, peripheral vascular disease, chronic kidney disease, gastrointestinal ulcers, chronic gastritis, hyperlipidemia, and valvular disease. Laboratory parameters recorded upon admission were white blood cell count, red blood cell count, platelet count, hemoglobin, hematocrit, alanine aminotransferase, aspartate aminotransferase, total bilirubin, albumin, blood urea nitrogen, serum creatinine, prothrombin time, activated partial thromboplastin time, international normalized ratio, and lactate dehydrogenase. Echocardiographic ejection fraction at admission was also documented. Definitions for each variable are provided in Supplementary Table S1.

Baseline blood glucose and glycated hemoglobin (HbA1c) were obtained from the first laboratory assessment after hospital admission. The SHR was calculated using the formula: SHR = blood glucose (mg/dL)/[28.7 * HbA1c (%) − 46.7]. Additionally, patients who underwent CABG were identified from the MIMIC-IV database using ICD-9/10 procedure codes, and comorbidities were identified using ICD-9/10 diagnosis codes (Supplementary Table S2). In the MIMIC-IV dataset, gastrointestinal bleeding events were identified using the information available within the database, including diagnosis codes, occult blood test results, and endoscopic records. Compared with the hospital cohorts, the MIMIC-IV dataset lacked the clinical manifestation information required by the predefined outcome definition (e.g., hematemesis, melena, and hematochezia). Therefore, standardized outcome adjudication according to the same predefined clinical criteria could not be performed for all cases.

### Statistical analysis

Data analysis was conducted using SPSS Statistical Software (version 26·0) and R (version 4·1·0). Continuous variables were summarized as means with standard deviations for those following a normal distribution, and as medians with interquartile ranges for skewed distributions. Independent samples t-tests were used to compare normally distributed continuous variables between two groups, while the Mann–Whitney U test was applied for non-normally distributed data. For comparisons across multiple groups, ANOVA was used for normally distributed data, and the Kruskal–Wallis test for non-normally distributed data. Categorical variables were reported as frequencies and percentages, with comparisons made using the chi-square test or Fisher' s exact test. The discriminatory ability of SHR, blood glucose, and HbA1c in predicting outcomes was evaluated using the area under the receiver operating characteristic curve (AUC). Comparisons of AUCs were performed using the DeLong test. Missing data were handled using multiple imputation techniques, with details regarding the methodology and parameters provided in the Supplementary Methods.

To explore the relationships between different SHR levels and the probabilities of GIBCG and in-hospital mortality, multivariate logistic regression models were employed. Additionally, restricted cubic spline (RCS) curves were used to further explore the relationship between changes in SHR and the risks of GIBCG and mortality. The multivariate logistic regression models were adjusted for relevant baseline factors. Variables with variance inflation factors greater than 10 were excluded. The adjustment factors used in the models for assessing GIBCG and in-hospital mortality are presented in detail in Supplementary Table S3.

In addition, subgroup analyses were performed to further explore the data. The study population was stratified by age, sex, and comorbidities (e.g., type 2 diabetes, hypertension, heart failure) (34). Moreover, subgroup analyses were conducted after excluding patients with a previous history of GIB. The differences in baseline characteristics among patients were also analyzed across subgroups stratified by year of surgery. Both univariate and multivariate logistic regression analyses were performed for each subgroup, adjusting for baseline factors as listed in Supplementary Table S3, but without including subgroup-defining variables. For example, sex was not adjusted in analyses for male and female subgroups. Age was categorized into two groups: <65 years and ≥65 years.

Sensitivity analyses were conducted to assess the robustness of the findings. First, all primary analyses were repeated after excluding the MIMIC-IV cohort. Second, in the full-cohort analyses, study center was included as a fixed-effect covariate in the multivariable logistic regression and restricted cubic spline models to account for potential between-center heterogeneity.

To evaluate the robustness of the primary findings and address the potential clinical heterogeneity introduced by the broad outcome definition, we performed a sensitivity analysis using clinically significant overt GIB as the outcome. Overt GIB was defined as bleeding requiring blood transfusion, endoscopic hemostasis, or associated with hemodynamic compromise. The same multivariable logistic regression models and restricted cubic spline analyses used in the primary analysis were applied in the sensitivity analysis.

## Results

### Patient characteristics

A total of 16,440 patients from five centers were included in the analysis. The extent of missing values and data types for each variable are detailed in Supplementary Table S4. To evaluate the impact of imputation on sample characteristics, a difference test was conducted between the datasets before and after imputation (Supplementary Table S5). Statistical analysis indicated no significant differences between them. The baseline characteristics of the study population are summarized in Table 1. The median age was 64 years (interquartile range: 13), and 4,213 (25.63%) were female. Among the patients, 6,246 (37.99%) had type 2 diabetes, 8,060 (49.03%) had hypertension, 6,374 (38.77%) had heart failure, and 860 (5.23%) had chronic kidney disease. The incidence of GIBCG was 7.04% (1,158/16,440), and the in-hospital mortality rate was 1.3% (214/16,440). Patients were divided into four groups based on quartiles of their SHR values: Group 1 (0.216–0.706), Group 2 (0.706–0.792), Group 3 (0.792–0.906), and Group 4 (0.906–2.902), each group consisting of 4,110 individuals. Group 2 had the highest proportion of females and lower prevalence rates of anemia, coagulation disorders, diabetes, and chronic kidney disease. This group also exhibited lower levels of white blood cells, red blood cells, urea, and creatinine compared to the other groups. This study included five centers, and Supplementary Table S6 presents the patient volumes and baseline characteristics for each center. The median sampling-to-surgery interval was 0.876 days (IQR, 0.239) in the overall cohort. No significant difference was observed between patients with and without postoperative GIB [0.878 [IQR, 0.246] vs. 0.875 [IQR, 0.239] days; *P* = 0.06; Supplementary Table S7].

**Table 1:** Patient demographics and baseline characteristics.

Characteristic	Stress hyperglycemia ratio					*p* value
	Overall, *N* = 16,440	Quartile 1, *N* = 4,110	Quartile 2, *N* = 4,110	Quartile 3, *N* = 4,110	Quartile 4, *N* = 4,110	
Demographics						
Age, median (IQR), y	64 (13)	63 (13)	65 (13)	65 (12)	64 (12)	<**0.001**
Sex, *n* (%)						**0**.**001**
Male	12,227 (74.37%)	3,156 (76.79%)	3,023 (73.55%)	3,024 (73.58%)	3,024 (73.58%)	
Female	4,213 (25.63%)	954 (23.21%)	1,087 (26.45%)	1,086 (26.42%)	1,086 (26.42%)	
Body mass index, median (IQR)	25.40 (4.24)	25.26 (4.35)	25.39 (4.24)	25.39 (4.24)	25.71 (4.15)	<**0**.**001**
Gastrointestinal bleeding history, *n* (%)	161 (0.98%)	33 (0.8%)	30 (0.73%)	43 (1.05%)	55 (1.34%)	**0**.**022**
After percutaneous coronary intervention, *n* (%)	1,868 (11.36%)	436 (10.61%)	451 (10.97%)	437 (10.63%)	544 (13.24%)	<**0**.**001**
Chronic comorbidities						
Anemia, *n* (%)	1,942 (11.81%)	502 (12.21%)	408 (9.93%)	439 (10.68%)	593 (14.43%)	<**0**.**001**
Coagulation disorders, *n* (%)	256 (1.56%)	47 (1.14%)	39 (0.95%)	52 (1.27%)	118 (2.87%)	<**0**.**001**
Hypertension, *n* (%)	8,060 (49.03%)	1,928 (46.91%)	2,086 (50.75%)	2,070 (50.36%)	1,976 (48.08%)	**0**.**001**
Atrial fibrillation, *n* (%)	1,061 (6.45%)	235 (5.72%)	258 (6.28%)	259 (6.3%)	309 (7.52%)	**0**.**008**
Diabetes, *n* (%)	6,246 (37.99%)	1,348 (32.8%)	1,246 (30.32%)	1,462 (35.57%)	2,190 (53.28%)	<**0**.**001**
Heart failure, *n* (%)	6,374 (38.77%)	1,571 (38.22%)	1,596 (38.83%)	1,546 (37.62%)	1,661 (40.41%)	0.057
Cerebral vascular disease, *n* (%)	2,582 (15.71%)	619 (15.06%)	635 (15.45%)	656 (15.96%)	672 (16.35%)	0.394
Peripheral vascular disease, *n* (%)	614 (3.73%)	143 (3.48%)	138 (3.36%)	158 (3.84%)	175 (4.26%)	0.131
Chronic kidney disease, *n* (%)	860 (5.23%)	207 (5.04%)	166 (4.04%)	183 (4.45%)	304 (7.4%)	<**0**.**001**
Gastrointestinal ulcer, *n* (%)	257 (1.56%)	52 (1.27%)	61 (1.48%)	62 (1.51%)	82 (2%)	0.055
Gastritis, *n* (%)	250 (1.52%)	43 (1.05%)	72 (1.75%)	68 (1.65%)	67 (1.63%)	**0**.**037**
Hyperlipidemia, *n* (%)	10,303 (62.67%)	2,502 (60.88%)	2,530 (61.56%)	2,552 (62.09%)	2,719 (66.16%)	<**0**.**001**
Valvular disease, *n* (%)	3,125 (19.01%)	791 (19.25%)	847 (20.61%)	838 (20.39%)	649 (15.79%)	<**0**.**001**
Admission examination						
White blood cell count, median (IQR), ×10^9^/L	6.8 (2.84)	6.79 (2.83)	6.67 (2.71)	6.69 (2.64)	7.12 (3.14)	<**0**.**001**
Red blood cell count, median (IQR), ×10^12^/L	4.39 (0.77)	4.42 (0.76)	4.41 (0.75)	4.41 (0.76)	4.32 (0.86)	<**0**.**001**
Platelet count, median (IQR), ×10^9^/L	204 (79)	206 (78)	204 (78)	204 (78)	203 (83)	**0**.**002**
Hemoglobin, median (IQR), g/L	135 (25)	137 (24)	136 (24)	136 (24)	133 (28)	<**0**.**001**
Hematocrit, median (IQR), Proportion of 1.0	0.395 (0.068)	0.398 (0.067)	0.397 (0.065)	0.397 (0.065)	0.387 (0.078)	<**0**.**001**
Alanine aminotransferase, median (IQR), µkat/L	0.351 (0.301)	0.351 (0.301)	0.351 (0.294)	0.341 (0.316)	0.343 (0.301)	0.409
Aspartate aminotransferase, median (IQR), µkat/L	0.334 (0.184)	0.317 (0.167)	0.329 (0.167)	0.334 (0.184)	0.334 (0.184)	**0**.**019**
Bilirubin total, median (IQR), µmol/L	11 (6.56)	10.9 (6.38)	11 (6.57)	11.06 (6.5)	11.01 (6.81)	0.091
Albumin, median (IQR), g/L	42 (5.1)	42 (4.7)	42 (4.9)	41.8 (5.2)	42.4 (5.5)	**0**.**003**
Urea, median (IQR), mmol/L	5.84 (2.39)	5.8 (2.23)	5.74 (2.33)	5.81 (2.38)	6.02 (2.57)	<**0**.**001**
Creatinine, median (IQR), μmol/L	74.7 (22.8)	75 (22.38)	73.7 (21.2)	73.9 (22.2)	76.3 (25)	<**0**.**001**
Prothrombin time, median (IQR), s	11.4 (1.1)	11.4 (1.1)	11.4 (1.1)	11.4 (1.1)	11.4 (1.3)	<**0**.**001**
Activated partial thromboplastin time, median (IQR), s	30.6 (4.7)	30.8 (4.58)	30.4 (4.8)	30.3 (4.9)	30.8 (4.5)	<**0**.**001**
International normalized ratio, median (IQR)	1.01 (0.09)	1.01 (0.09)	1.01 (0.1)	1.01 (0.1)	1.02 (0.11)	<**0**.**001**
Lactate dehydrogenase, median (IQR), µkat/L	2.99 (0.87)	2.97 (0.79)	2.99 (0.87)	3.01 (0.87)	3.01 (0.94)	**0**.**022**
Ejection fraction, No. (%)						**0**.**002**
≥55%	12,292 (74.77%)	3,152 (76.69%)	3,081 (74.96%)	3,023 (73.55%)	3,036 (73.87%)	
45–55%	2,427 (14.76%)	581 (14.14%)	590 (14.36%)	638 (15.52%)	618 (15.04%)	
30–45%	1,583 (9.63%)	356 (8.66%)	402 (9.78%)	420 (10.22%)	405 (9.85%)	
<30%	138 (0.84%)	21 (0.51%)	37 (0.9%)	29 (0.71%)	51 (1.24%)	
Antithrombotic medications						
Single antiplatelet therapy, *n* (%)	2,862 (17.41%)	686 (16.69%)	709 (17.25%)	742 (18.05%)	725 (17.64%)	0.41
Dual antiplatelet therapy, *n* (%)	3,671 (22.33%)	754 (18.35%)	917 (22.31%)	947 (23.04%)	1,053 (25.62%)	<**0**.**001**
Oral anticoagulants, *n* (%)	153 (0.93%)	38 (0.92%)	38 (0.92%)	29 (0.71%)	48 (1.17%)	0.189
Surgical characteristics						
Surgical duration, median (IQR), h	4.25 (1.33)	4.25 (1.28)	4.25 (1.50)	4.25 (1.33)	4.28 (1.36)	0.11
No. of grafts, median (IQR)	2.78 (1.11)	2.78 (1.11)	2.75 (1.10)	2.78 (1.10)	2.81 (1.11)	0.076
On pump, *n* (%)	8,250 (50.18%)	2,042 (49.68%)	2,048 (49.83%)	2,040 (49.64%)	2,120 (51.58%)	0.229
On pump time, median (IQR), min	158 (83.3)	159 (83)	161 (84)	159 (83)	156 (80)	0.871
Aortic occlusion time, median (IQR), min	91 (80)	90 (79)	95 (81)	96 (83)	88 (72)	0.649
Incidence of GIBCG, *n* (%)	1,158 (7.04%)	217 (5.28%)	173 (4.21%)	223 (5.43%)	545 (13.26%)	<**0**.**001**
In-hospital mortality, *n* (%)	214 (1.3%)	49 (1.19%)	30 (0.73%)	52 (1.27%)	83 (2.02%)	<**0**.**001**

SHR: Quartile 1 (0.216–0.706), Quartile 2 (0.706–0.792), Quartile 3 (0.792–0.906), and Quartile 4 (0.906–2.902).

### Association between SHR and GIBCG

**Figure 2:**
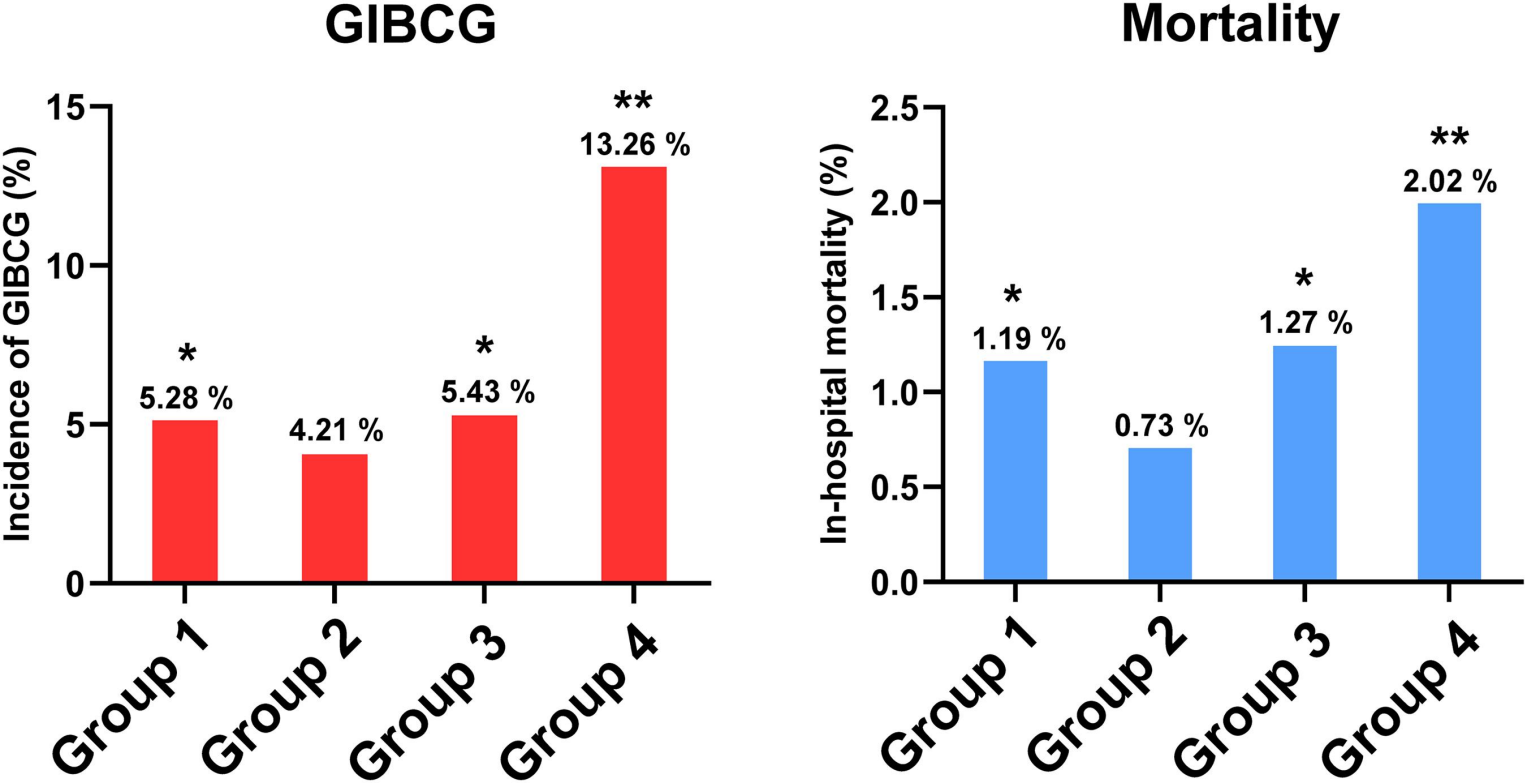
Incidence comparison of outcome events Among four groups. The left panel displays the incidence of GIBCG across four groups, while the right panel presents the corresponding in-hospital mortality rates. *P < 0.05 and **P < 0.001 indicate statistically significant differences compared with Group 2.

**Figure 3:**
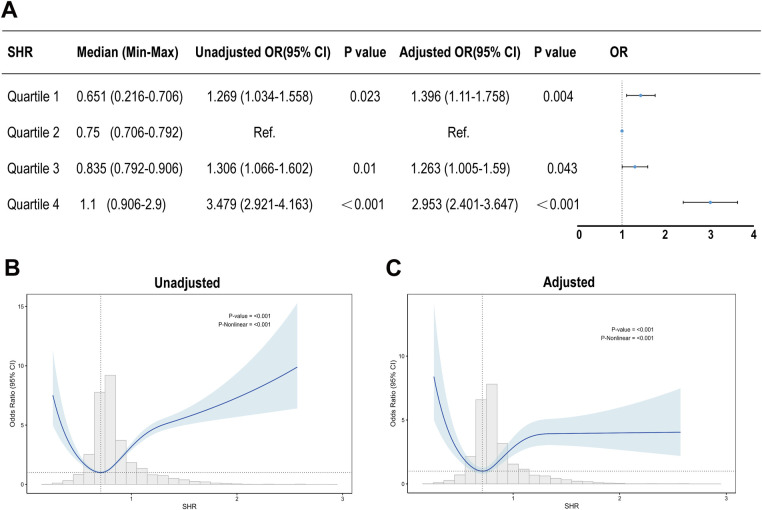
Relationship between stress hyperglycemia ratio and gastrointestinal bleeding after coronary artery bypass grafting. **(A)** Comparison of the risk of GIBCG among patients with different SHR levels. **(B)** Restricted cubic spline: A U-shaped relationship between SHR and GIBCG in the unadjusted model. **(C)** Restricted cubic spline: The U-shaped relationship persisted in the adjusted model.

Figure 2 presents the incidence of adverse outcomes across four SHR groups. Group 2 exhibited the lowest incidence of GIBCG, at 4.21%, as well as the lowest in-hospital mortality rate of 0.73%. Consequently, Group 2 was selected as the reference group for comparison. In the unadjusted model (Figure 3A), the risk of GIBCG was higher in the other groups compared to Group 2. The odds ratio (OR) were 1.269 (95% CI: 1.034–1.558, *P* = 0.023) for Group 1, 1.306 (95% CI: 1.066–1.602, *P* = 0.01) for Group 3, and 3.479 (95% CI: 2.921–4.163, *P* < 0.001) for Group 4. After adjusting for confounders, the U-shaped relationship persisted, with adjusted OR of 1.396 (95% CI: 1.11–1.758, *P* = 0.004) for Group 1, 1.263 (95% CI: 1.005–1.59, *P* = 0.043) for Group 3, and 2.953 (95% CI: 2.401–3.647, *P* < 0.001) for Group 4. Additionally, an RCS analysis was performed to examine the relationship between SHR and GIBCG across its entire spectrum. Both the unadjusted (Figure 3B) and adjusted models (Figure 3C) confirmed a U-shaped association, with the lowest risk of GIBCG observed at an SHR of 0.71, within the reference range of 0.706–0.792.

### Association between SHR and in-hospital mortality

**Figure 4:**
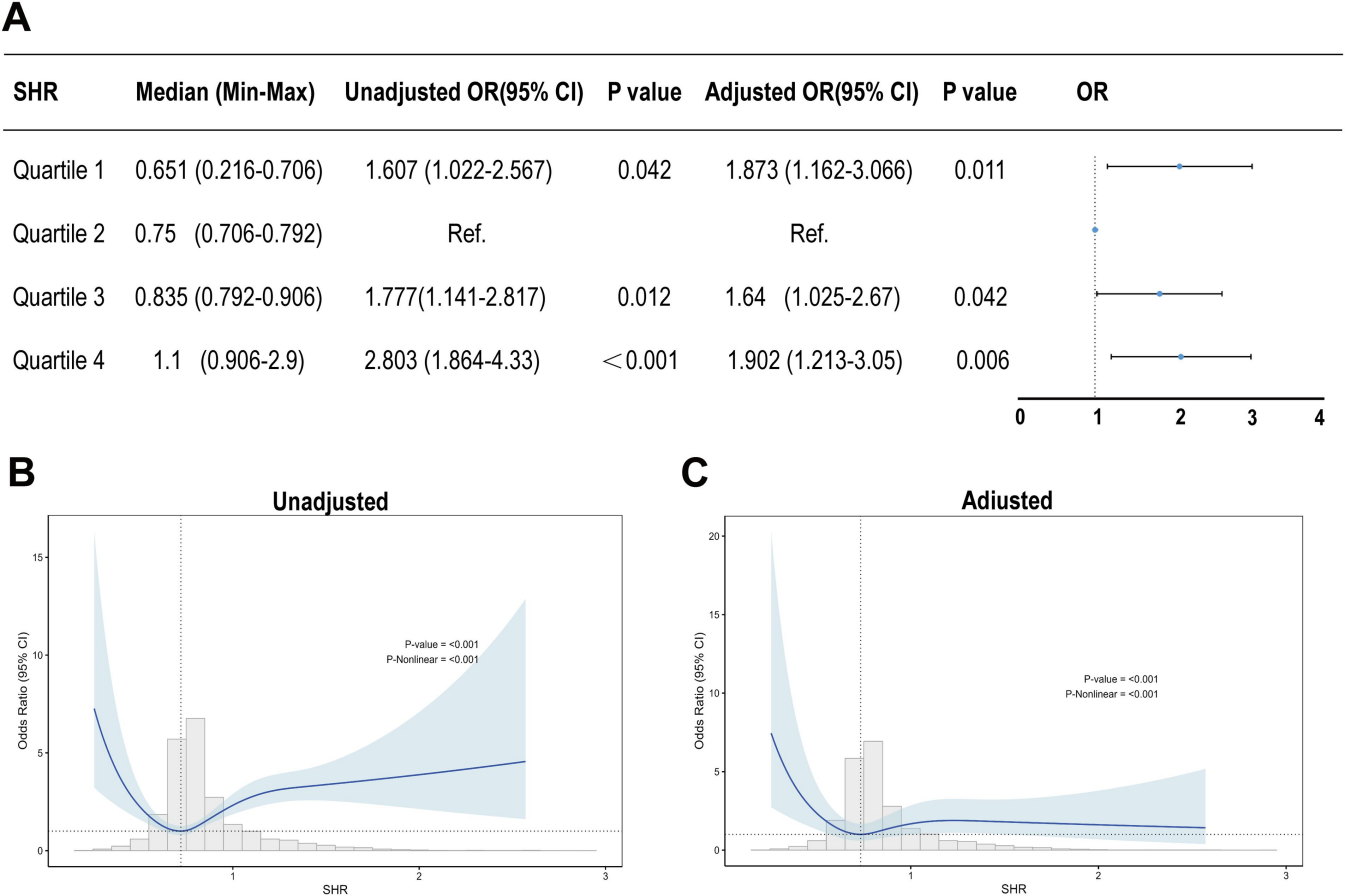
Relationship between stress hyperglycemia ratio and in-hospital mortality in patients undergoing coronary artery bypass grafting. **(A)** Comparison of in-hospital mortality risk among patients with different SHR levels. **(B)** Restricted cubic spline: A U-shaped relationship between SHR and in-hospital mortality in the unadjusted model. **(C)** Restricted cubic spline: The U-shaped relationship persisted in the adjusted model.

The association between SHR and in-hospital mortality followed a similar trend. In the unadjusted model (Figure 4A), in-hospital mortality was higher in the other groups compared to Group 2. The OR was 1.607 (95% CI: 1.022–2.567, *P* = 0.042) for Group 1, 1.777 (95% CI: 1.141–2.817, *P* = 0.012) for Group 3, and 2.803 (95% CI: 1.864–4.330, *P* < 0.001) for Group 4. After adjustment, the OR was 1.873 (95% CI: 1.162–3.066, *P* = 0.011) for Group 1, 1.64 (95% CI: 1.025–2.67, *P* = 0.042) for Group 3, and 1.902 (95% CI: 1.213–3.05, *P* = 0.006) for Group 4. Figures 4B,C demonstrated a consistent U-shaped relationship between SHR and in-hospital mortality, regardless of model adjustments. In the adjusted model, the lowest risk of in-hospital mortality was observed at an SHR of approximately 0.733.

### Subgroup analysis

Comprehensive investigation into the complex associations between the SHR and both the risk of GIBCG and in-hospital mortality was conducted across diverse subgroups. A consistent U-shaped association between SHR and the risk of GIBCG emerged in all subgroups, regardless of adjustment for confounding factors (Table 2). A similar U-shaped relationship between SHR and in-hospital mortality was also observed across all subgroups (Table 3). Notably, patients in the highest quartile of SHR consistently exhibited significantly increased risks of both GIBCG and in-hospital death. Furthermore, among patients without diabetes, both elevated and reduced SHR levels were associated with higher risks of GIBCG and in-hospital mortality compared to those with diabetes. In addition, we expanded our investigation by conducting meticulous RCS analyses within these subgroups, which further confirmed the robustness of the U-shaped association between SHR and GIBCG in both the unadjusted (Supplementary Figure S1) and adjusted models (Supplementary Figure S2). Similarly, the U-shaped relationship between SHR and in-hospital mortality was consistently observed across all subgroups in both the unadjusted (Supplementary Figure S3) and adjusted models (Supplementary Figure S4). It is worth noting that we additionally reported the number of in-hospital deaths across all subgroups in Supplementary Table S8. Given the relatively small number of events in several subgroups, a simplified, pre-specified set of key clinical covariates was used for confounder adjustment in the subgroup analyses of in-hospital mortality (Supplementary Table S3).

**Table 2:** Subgroup analysis assessing the association between SHR and GIBCG through odds ratios.

SHR	Unadjusted OR (95%CI)	Unadjusted *P* value	Adjusted OR (95%CI)	Adjusted *P* value
Age ≥ 65 (*n* = 8,008)				
Quartile 1	1.324 (1.012–1.737)	0.041	1.498 (1.106–2.037)	0.009
Quartile 2	Reference		Reference	
Quartile 3	1.335 (1.021–1.75)	0.035	1.406 (1.036–1.913)	0.029
Quartile 4	4.3 (3.425–5.446)	<0.001	3.68 (2.803–4.872)	<0.001
Age<65 (*n* = 8,432)				
Quartile 1	1.428 (1.038–1.976)	0.030	1.451 (1.017–2.082)	0.041
Quartile 2	Reference		Reference	
Quartile 3	1.38 (1–1.913)	0.051	1.202 (0.838–1.732)	0.320
Quartile 4	2.976 (2.244–3.995)	<0.001	2.42 (1.74–3.4)	<0.001
Female (*n* = 4,213)				
Quartile 1	1.974 (1.309–3.029)	0.001	2.508 (1.556–4.122)	<0.001
Quartile 2	Reference		Reference	
Quartile 3	1.542 (1.002–2.401)	0.052	1.676 (1.019–2.795)	0.044
Quartile 4	4.757 (3.295–7.05)	<0.001	4.771 (3.055–7.653)	<0.001
Male (*n* = 12,227)				
Quartile 1	1.196 (0.937–1.531)	0.152	1.314 (1.002–1.725)	0.049
Quartile 2	Reference		Reference	
Quartile 3	1.607 (1.277–2.031)	<0.001	1.64 (1.269–2.127)	<0.001
Quartile 4	3.551 (2.891–4.392)	<0.001	3.007 (2.367–3.842)	0.001
Diabetes_II_Yes (*n* = 6,246)				
Quartile 1	1.247 (0.891–1.752)	0.200	1.285 (0.879–1.888)	0.198
Quartile 2	Reference		Reference	
Quartile 3	1.896 (1.392–2.607)	<0.001	2.082 (1.461–2.996)	<0.001
Quartile 4	3.166 (2.375–4.274)	<0.001	3.058 (2.186–4.333)	<0.001
Diabetes_II_No (*n* = 10,194)				
Quartile 1	1.815 (1.38–2.402)	<0.001	1.989 (1.47–2.709)	<0.001
Quartile 2	Reference		Reference	
Quartile 3	1.474 (1.108–1.969)	0.008	1.419 (1.035–1.955)	0.031
Quartile 4	5.487 (4.319–7.052)	<0.001	4.243 (3.228–5.638)	<0.001
Hypertension_Yes (*n* = 8,060)				
Quartile 1	1.515 (1.123–2.054)	0.007	1.8 (1.286–2.535)	0.001
Quartile 2	Reference		Reference	
Quartile 3	1.413 (1.043–1.923)	0.026	1.363 (0.967–1.929)	0.079
Quartile 4	4.696 (3.635–6.144)	<0.001	4.336 (3.203–5.937)	<0.001
Hypertension_No (*n* = 8,380)				
Quartile 1	1.328 (0.999–1.771)	0.052	1.385 (1.003–1.917)	0.049
Quartile 2	Reference		Reference	
Quartile 3	1.44 (1.088–1.912)	0.011	1.5 (1.095–2.064)	0.012
Quartile 4	3.014 (2.35–3.901)	<0.001	2.367 (1.755–3.216)	<0.001
Heart_Failure_Yes (*n* = 6,374)				
Quartile 1	1.277 (0.976–1.675)	0.076	1.446 (1.066–1.966)	0.018
Quartile 2	Reference		Reference	
Quartile 3	1.498 (1.154–1.952)	0.003	1.495 (1.112–2.016)	0.008
Quartile 4	3.52 (2.79–4.475)	<0.001	3.301 (2.505–4.38)	<0.001
Heart_Failure_No (*n* = 10,066)				
Quartile 1	1.504 (1.098–2.074)	0.012	1.605 (1.133–2.289)	0.008
Quartile 2	Reference		Reference	
Quartile 3	1.267 (0.914–1.763)	0.158	1.167 (0.813–1.683)	0.404
Quartile 4	3.701 (2.815–4.93)	<0.001	2.672 (1.937–3.727)	<0.001

**Table 3:** Subgroup analysis assessing the association between SHR and in-hospital mortality through odds ratios.

SHR	Unadjusted OR (95%CI)	Unadjusted *P* value	Adjusted OR (95%CI)	Adjusted *P* value
Age ≥ 65 (*n* = 8,008)				
Quartile 1	1.776 (1.046–3.096)	0.037	1.878 (1.097–3.297)	0.024
Quartile 2	Reference		Reference	
Quartile 3	1.679 (0.982–2.939)	0.062	1.717 (0.998–3.025)	0.055
Quartile 4	3.065 (1.896–5.159)	<0.001	2.151 (1.302–3.684)	0.004
Age < 65 (*n* = 8,432)				
Quartile 1	2.006 (0.777–5.771)	0.165	2.167 (0.819–6.389)	0.132
Quartile 2	Reference		Reference	
Quartile 3	3.017 (1.262–8.336)	0.019	3.081 (1.259–8.693)	0.020
Quartile 4	3.695 (1.59–10.05)	0.005	3.162 (1.317–8.832)	0.016
Female (*n* = 4,213)				0.000
Quartile 1	2.655 (1.216–6.406)	0.019	3.188 (1.415–7.919)	0.008
Quartile 2	Reference		Reference	
Quartile 3	1.76 (0.751–4.423)	0.204	1.931 (0.808–4.948)	0.149
Quartile 4	3.047 (1.421–7.272)	0.007	2.575 (1.158–6.325)	0.027
Male (*n* = 12,227)				
Quartile 1	1.772 (0.987–3.288)	0.060	2.019 (1.112–3.786)	0.024
Quartile 2	Reference		Reference	
Quartile 3	2.371 (1.365–4.3)	0.003	2.443 (1.391–4.476)	0.003
Quartile 4	3.703 (2.212–6.547)	<0.001	2.863 (1.667–5.164)	0.000
Diabetes_II_Yes (*n* = 6,246)				
Quartile 1	1.781 (0.912–3.629)	0.098	2.103 (1.063–4.656)	0.042
Quartile 2	Reference		Reference	
Quartile 3	1.468 (0.729–3.052)	0.288	1.653 (0.802–3.517)	0.178
Quartile 4	2.573 (1.382–5.086)	0.004	2.124 (1.107–4.306)	0.028
Diabetes_II_No (*n* = 10,194)				
Quartile 1	2.48 (1.329–4.906)	0.006	2.512 (1.332–5.017)	0.006
Quartile 2	Reference		Reference	
Quartile 3	2.403 (1.283–4.764)	0.008	2.036 (1.077–4.069)	0.034
Quartile 4	3.905 (2.183–7.513)	<0.001	2.524 (1.368–4.967)	0.005
Hypertension_Yes (*n* = 8,060)				
Quartile 1	1.743 (0.932–3.379)	0.088	2.088 (1.098–4.115)	0.028
Quartile 2	Reference		Reference	
Quartile 3	1.947 (1.057–3.738)	0.037	1.968 (1.054–3.824)	0.038
Quartile 4	3.602 (2.077–6.638)	<0.001	2.97 (1.664–5.604)	0.000
Hypertension_No (*n* = 8,380)				
Quartile 1	3.151 (1.481–7.482)	0.005	3.643 (1.686–8.766)	0.002
Quartile 2	Reference		Reference	
Quartile 3	3.278 (1.548–7.763)	0.003	3.651 (1.7–8.749)	0.002
Quartile 4	4.047 (1.955–9.454)	<0.001	2.822 (1.317–6.753)	0.012
Heart_Failure_Yes (*n* = 6,374)				
Quartile 1	1.892 (1.041–3.567)	0.041	2.186 (1.18–4.197)	0.015
Quartile 2	Reference		Reference	
Quartile 3	2.41 (1.364–4.461)	0.003	2.435 (1.354–4.577)	0.004
Quartile 4	3.064 (1.772–5.587)	<0.001	2.151 (1.199–4.039)	0.013
Heart_Failure_No (*n* = 10,066)				
Quartile 1	2.345 (1.105–5.4)	0.033	2.567 (1.196–5.977)	0.020
Quartile 2	Reference		Reference	
Quartile 3	2.345 (1.105–5.403)	0.033	2.401 (1.123–5.574)	0.030
Quartile 4	3.476 (1.722–7.772)	0.001	2.896 (1.403–6.581)	0.006

Prior GIB is also an important baseline risk factor. To eliminate its potential confounding effect, we conducted a subgroup analysis excluding patients with prior GIB. Supplementary Figure S5 illustrates the association between SHR and GIBCG, with Supplementary Figure S5A showing that the risk of GIBCG was higher in the other groups compared to Group 2. Both the unadjusted (Supplementary Figure S5B) and adjusted models (Supplementary Figure S5C) confirmed a U-shaped association. Similarly, Supplementary Figure S6 demonstrates that the U-shaped association between SHR and in-hospital mortality persists.

Furthermore, patients were stratified by surgical year for subgroup analysis, with the results presented in Supplementary Table S9. No statistically significant differences were observed across the surgical year subgroups in terms of demographic characteristics, major comorbidities (such as hypertension and diabetes), admission examination parameters (including SHR and ejection fraction), the incidence of GIBCG, or in-hospital mortality. These findings suggest a balanced distribution of baseline characteristics and outcomes among patients across different surgical years.

The study included patients from four medical centers and the MIMIC-IV cohort. After excluding the MIMIC-IV cohort, the U-shaped associations between SHR and GIBCG and between SHR and in-hospital mortality remained (Supplementary Figures S7, S8). In the full-cohort analysis, after adjustment for study center as a fixed-effect covariate, the risks of GIBCG and in-hospital mortality remained higher in Groups 1, 3, and 4 than in Group 2 (Supplementary Table S10). Similarly, after adjustment for study center in the restricted cubic spline models, the U-shaped associations between SHR and GIBCG and between SHR and in-hospital mortality persisted (Supplementary Figure S9).

A sensitivity analysis was also performed using clinically significant overt gastrointestinal bleeding as the outcome. The proportion of overt gastrointestinal bleeding events among all gastrointestinal bleeding events is presented in Supplementary Table S11. Multivariable logistic regression analysis showed that both low and high SHR levels were associated with an increased risk of overt gastrointestinal bleeding, with an overall pattern of association consistent with the primary analysis (Supplementary Figure S10). Restricted cubic spline analysis further demonstrated a U-shaped association between SHR and the risk of overt gastrointestinal bleeding (Supplementary Figure S10).

### Comparison of the predictive value of SHR and other indicators

The predictive performance of SHR and other indicators for GIBCG and in-hospital mortality was evaluated based on the AUC. Supplementary Figure S11 presents the ROC curve analysis for SHR and other indicators in predicting GIBCG. The AUC for SHR was 0.677 [95% confidence interval (CI), 0.659–0.694], significantly outperforming blood glucose (AUC = 0.632, 95% CI: 0.615–0.649; *P* < 0.001), HbA1c (AUC = 0.548, 95% CI: 0.529–0.567; *P* < 0.001), and the presence of diabetes mellitus (AUC = 0.524, 95% CI: 0.510–0.538; *P* < 0.001). Supplementary Figure S12 shows the ROC curve analysis for SHR and other indicators in predicting in-hospital mortality. SHR achieved an AUC of 0.696 (95% CI: 0.657–0.737), again significantly superior to blood glucose (AUC = 0.637, 95% CI: 0.596–0.677; *P* < 0.001), HbA1c (AUC = 0.517, 95% CI: 0.474–0.563; *P* < 0.001), and the presence of diabetes mellitus (AUC = 0.509, 95% CI: 0.477–0.543; *P* < 0.001).

## Discussion

In this multicenter study, we identified a U-shaped relationship between admission SHR and both GIBCG incidence and in-hospital mortality in patients undergoing CABG surgery. Specifically, the risk of GIBCG was lowest at an SHR of approximately 0.71, while in-hospital mortality reached its minimum at an SHR of around 0.733. Deviations from these optimal SHR levels, whether lower or higher, were associated with an increased risk of both GIBCG and mortality. Furthermore, these findings suggest that SHR may serve as a useful marker for preoperative risk stratification in patients undergoing CABG, although whether SHR-guided interventions can improve clinical outcomes requires further prospective validation.

Our study included patients from four medical centers and the MIMIC-IV database, thereby increasing the heterogeneity of the study population and supporting the generalizability of our findings. We acknowledge that differences in data structure, case mix, and outcome ascertainment between MIMIC-IV and multicenter hospital cohorts may introduce heterogeneity in outcome ascertainment and absolute event rates. Specifically, gastrointestinal bleeding events in the MIMIC-IV database were identified using diagnosis codes, occult blood test results, and endoscopic records, whereas standardized outcome adjudication in the hospital cohorts additionally relied on predefined clinical manifestations. Consequently, outcome ascertainment was not fully harmonized across data sources. In addition, besides the greater severity of illness in the MIMIC-IV cohort, differences in outcome ascertainment may also have contributed, at least in part, to the higher incidence of GIBCG observed in this cohort. However, sensitivity analyses demonstrated that the U-shaped associations of SHR with both GIBCG and in-hospital mortality remained unchanged after excluding the MIMIC-IV cohort or after adjustment for study center. aken together, these findings indicate that the observed associations are robust and not materially influenced by the inclusion of the MIMIC-IV cohort or inter-center differences, and were consistently supported by analyses across different data sources and modeling strategies.

The primary outcome included patients with positive fecal or gastric occult blood tests. Although occult blood positivity alone does not necessarily indicate clinically significant overt gastrointestinal bleeding, it may reflect gastrointestinal mucosal injury or subclinical bleeding. Therefore, we adopted a broader outcome definition to evaluate the predictive value of the SHR for the early risk of gastrointestinal bleeding rather than limiting the outcome to established severe bleeding events. To further evaluate the robustness of our findings, we performed a sensitivity analysis using clinically significant overt gastrointestinal bleeding as the outcome. The sensitivity analysis demonstrated a similar U-shaped association between SHR and overt gastrointestinal bleeding, consistent with the primary analysis. These findings support the robustness of our primary results and suggest that SHR may have potential value in identifying patients at increased risk of both early and clinically significant gastrointestinal bleeding.

The Stress Hyperglycemia Ratio (SHR) is a novel metabolic marker that improves the assessment of glycemic changes during acute physiological stress, such as severe infection, trauma, or surgery (10, 11). Unlike traditional measures such as preoperative blood glucose, HbA1c, or diabetes history, SHR provides a more accurate and context-specific evaluation. Conventional indicators often fail to distinguish stress-induced hyperglycemia from chronic hyperglycemia, limiting their usefulness in acute clinical settings. SHR accounts for a patient's baseline glycemic status, as estimated by HbA1c, to adjust acute blood glucose levels. This correction minimizes the confounding effects of chronic hyperglycemia and provides a more precise reflection of stress-induced glycemic disturbances (35). Consequently, SHR better captures acute stress-related glycemic disturbances while accounting for baseline glycemic status, thereby separating them from long-standing metabolic patterns.

Numerous studies have demonstrated the prognostic value of SHR. Stress hyperglycemia has been linked to larger myocardial infarctions and a higher risk of adverse cardiovascular events, making it an independent predictor of poor outcomes in patients with acute coronary syndrome (36, 37). In a study of 5,562 patients, Yang et al. found a U-shaped association between the SHR and major adverse cardiovascular events during a two-year follow-up (11). In some patients with severe acute coronary syndromes, CABG is a key therapeutic tool to improve prognosis. Patients undergoing CABG often experience substantial physiological stress before surgery because of their underlying cardiovascular disease, while the surgical procedure itself further amplifies this response. Given that the median sampling-to-surgery interval in our cohort was only 0.876 days, SHR was measured shortly before surgery and is therefore likely to reflect the patients' immediate preoperative metabolic status. Previous studies have shown that elevated SHR in CABG patients correlates with increased in-hospital mortality and a higher incidence of complications, including myocardial infarction, stroke, and acute kidney injury (38). There is a U-shaped association between SHR and in-hospital events, and a linear positive correlation with long-term outcomes following CABG (34). Notably, stress ulcers caused by stress are one of the major risk factors for GIBCG, but the association between SHR and GIBCG is unclear. Using RCS analysis, our study is the first to demonstrate a U-shaped relationship between SHR and both GIBCG and in-hospital mortality in CABG patients, irrespective of adjustments for potential confounders. However, the underlying mechanisms of the U-shaped association between SHR and both GIBCG and in-hospital mortality remain unclear and may involve multiple physiological and metabolic factors.

Surgical stress activates the sympathetic nervous system and the hypothalamic-pituitary-adrenal axis, triggering the release of inflammatory cytokines and promoting insulin resistance, which contribute to stress hyperglycemia (10, 39). Stress hyperglycemia is considered an adaptive response that may exert short-term protective effects during ischemic states by sustaining energy supply (40). Moderate hyperglycemia can optimize glucose utilization (41), improve tissue oxygen delivery, reduce oxidative stress induced by lipid metabolism, and lower cardiovascular risks associated with hypoglycemia. Additionally, it may reduce myocardial infarction rates and enhance cardiac contractile function by upregulating the expression of vascular endothelial growth factor and hypoxia-inducible factor-1α (42).

SHR is a key prognostic marker, as both elevated and reduced levels may be associated with adverse clinical outcomes. A low SHR may indicate that the patient is in a state of chronic hyperglycemia, meaning that blood glucose levels have been high over a long period, but were overly controlled prior to surgery. This may increase the incidence of hypoglycemic events, which can excessively activate the sympathetic nervous system, leading to vasoconstriction, increased cardiac workload, and potentially fatal arrhythmias (43). Moreover, hypoglycemia-induced sympathetic overactivation may cause peripheral vasoconstriction to prioritize blood supply to vital organs such as the brain and heart, but at the same time may lead to insufficient gastrointestinal microcirculatory perfusion (44). In addition, suppression of parasympathetic function weakens the autonomic regulation of the gastrointestinal tract, affecting its normal peristalsis and secretion, further aggravating gastrointestinal dysfunction. An excessively high SHR may reflect an excessive stress response in patients, leading to persistent hyperglycemia. This state may weaken the gastric mucosal barrier through mechanisms such as sympathetic activation, insufficient gastrointestinal microcirculatory perfusion, release of pro-inflammatory cytokines (45), and increased gastric acid secretion, thereby inducing stress ulcers and increasing the risk of ulcer bleeding. Hyperglycemia not only exacerbates inflammatory responses and oxidative stress, but may also damage the vascular endothelium, enhance platelet aggregation, and disrupt coagulation function, thereby increasing the risk of postoperative thrombosis and ischemic events (46). However, persistent hyperglycemia impairs vascular barrier function and increases microvascular fragility (47). Although hyperglycemia promotes thrombosis, when it is prolonged and accompanied by significant endothelial injury (48), microvascular fragility increases, and combined with coagulation-fibrinolysis imbalance, it may instead raise the risk of bleeding events such as GIB. Therefore, perioperative blood glucose management in CABG patients should be optimized based on individual conditions to reduce the risk of related complications, while avoiding hypoglycemia or poor glycemic control. In the present study, deviations in SHR from the observed range of 0.706–0.792 were associated with increased risks of GIBCG and in-hospital mortality. These findings support the potential utility of SHR as a prognostic marker for perioperative risk stratification and identification of patients at increased risk. In clinical practice, SHR may help identify patients who warrant closer perioperative monitoring and individualized clinical assessment, particularly with regard to glycemic management and surveillance for postoperative gastrointestinal bleeding. Further prospective studies are warranted to determine whether SHR-guided management can improve clinical outcomes.

Previous studies have demonstrated an association between the SHR and major adverse cardiac events following CABG; however, these analyses were limited to non-diabetic patients only (38). This study further explored how glucose metabolic status modifies the individual associations between SHR and two clinical outcomes: GIBCG and in-hospital mortality. Notably, in non-diabetic patients, both markedly high and low SHR levels were linked to significantly increased risks of GIBCG and in-hospital mortality, compared to their diabetic counterparts. This difference has also been observed in previous research. One study reported a stronger association between SHR and mortality in non-diabetic patients than in those with diabetes (49). Similarly, Li et al. identified a U-shaped relationship between SHR and acute kidney injury in patients with congestive heart failure. Their subgroup analysis revealed that non-diabetic patients faced a relatively higher risk of in-hospital mortality than diabetic patients (50).

The more pronounced adverse effects of extreme SHR values in non-diabetic patients remain mechanistically unclear. One possible explanation is that individuals with diabetes may develop adaptive responses to the pathophysiological effects of hyperglycemia (51). Due to prolonged exposure to elevated glucose levels, these patients may activate compensatory mechanisms (10), including enhanced antioxidant production, downregulation of glucose transporters, upregulation of survival factors, and increased angiogenesis (42, 52, 53). These adaptations may enable the body to better tolerate the harmful effects of acute hyperglycemia. Additionally, insulin therapy in diabetic patients may exert anti-inflammatory and protective effects, thereby reducing the harm associated with stress hyperglycemia (54). These findings suggest that while moderate glycemic control may be sufficient for diabetic patients, non-diabetic patients may benefit from more tailored postoperative glucose management to mitigate the risks associated with stress hyperglycemia. Further large-scale prospective studies are warranted to validate the clinical utility of SHR in guiding glycemic strategies for CABG patients. In addition to diabetes, subgroup analyses were performed based on age, sex, and the presence of comorbidities such as hypertension, heart failure, and a history of gastrointestinal bleeding. The results consistently demonstrated a U-shaped association between the SHR and outcomes across all subgroups. This robust and consistent pattern underscores the stability of the relationship and further supports the predictive value of SHR in diverse patient populations.

### Limitations

This study has several limitations. First, this multicenter analysis included not only a prospective cohort but also partial retrospective cohorts, which may introduce selection bias. To mitigate this, we applied strict inclusion and exclusion criteria and enrolled a large sample size. Second, since the cohort focused solely on in-hospital mortality, we were unable to evaluate the long-term association between SHR and adverse outcomes. In addition, our analysis was primarily based on the SHR value at admission and did not account for dynamic changes in glucose levels throughout the perioperative period, which warrants further investigation. Moreover, although SHR outperformed admission glucose and HbA1c, its discriminatory ability remained moderate. Therefore, SHR should be considered a complementary marker for perioperative risk stratification rather than a standalone predictive tool. Future studies integrating SHR with additional clinical and perioperative variables may further improve predictive performance. Furthermore, although we adjusted for numerous known confounders, the observed associations may still have been influenced by unmeasured or unknown perioperative and postoperative factors that were unavailable in our dataset. These include factors such as perioperative transfusion requirements, vasopressor use, insulin therapy, stress-ulcer prophylaxis, and postoperative glycemic management, which were unavailable for adjustment in the present study. Their absence represents a limitation of this study and may have introduced residual confounding, potentially leading to either overestimation or underestimation of the observed effects. In addition, in the multivariable model for the mortality outcome, a relatively large number of covariates were included based on prior evidence and their clinical relevance to the mortality outcome. However, the events-per-variable remained at the lower boundary of the commonly recommended range. Therefore, the possibility of model instability and overfitting cannot be fully excluded. To address this issue, subgroup analyses were performed using a pre-specified, clinically driven simplified set of covariates. This approach aimed to improve model robustness under limited event conditions while reducing the risk of overfitting and maintaining adequate control for major confounders.

## Conclusions

SHR demonstrated a U-shaped association with both GIB and in-hospital mortality among patients undergoing CABG. The nadir of the observed U-shaped association occurred within the SHR range of 0.706–0.792, with inflection points at 0.71 for GIB and 0.733 for in-hospital mortality. Both low and high SHR levels were associated with an increased risk of GIB and in-hospital mortality. These findings suggest that SHR may serve as a useful marker for perioperative risk stratification in patients undergoing CABG. Further large-scale, multicenter prospective studies are warranted to validate its prognostic value and determine whether SHR-guided interventions can improve clinical outcomes.

## Data Availability

The raw data supporting the conclusions of this article will be made available by the authors, without undue reservation.
