# Supplementary material for: Relationship between stress hyperglycemia ratio and gastrointestinal bleeding and mortality after coronary artery bypass grafting: a multicenter cohort study
Source: Front Cardiovasc Med. 2026 Jul 22;13:1873683. doi: 10.3389/fcvm.2026.1873683 (PMC13437271; doi:10.3389/fcvm.2026.1873683)
Supplement: Supplementary file 1 [file Datasheet1.doc]

**Supplementary materials**

Supplementary Methods.	2

Table S1. The definition of each variable collected in this study	3

Table S2. ICD-9/ICD-10 Codes are Used to Identify Chronic Comorbidities and Procedures	6

Table S3. Confounding factors adjusted in the multivariate logistic regression models	9

Table S4. Description of the Data Types and Missing Values for Each Variable	10

Table S5. Comparison of Sample Characteristics Before and After Imputation	12

Table S6. Data Volume and Baseline Characteristics of Each Center	13

Table S7. Comparison of the SHR Sampling-to-Surgery Interval Between Patients With and Without Postoperative Gastrointestinal Bleeding	16

Table S8. Number of In-Hospital Deaths Across All Subgroups	17

Table S9 Demographic and Baseline Characteristics of Patients Stratified by Year of Surgery	18

Table S10. Associations Between Stress Hyperglycemia Ratio Quartiles and Clinical Outcomes After Adjustment for Study Center as a Fixed-Effect Covariate	20

Table S11. The proportion of overt gastrointestinal bleeding events among all gastrointestinal bleeding events.	21

Figure S1. Subgroup Analyses of the Association Between SHR and GIBCG in the Unadjusted Models.	22

Figure S2. Subgroup Analyses of the Association Between SHR and GIBCG in the Adjusted Models.	23

Figure S3. Subgroup Analyses of the Association Between SHR and In-Hospital Mortality in the Unadjusted Models.	25

Figure S4. Subgroup Analyses of the Association Between SHR and In-Hospital Mortality in the Adjusted Models.	26

Figure S5. Subgroup Analysis of the Association Between SHR and GIBCG in Patients Without a History of Gastrointestinal Bleeding	27

Figure S6. Subgroup analyses of the association between SHR and In-Hospital Mortality in Patients Without a History of Gastrointestinal Bleeding	28

Figure S7. Relationship Between the Stress Hyperglycemia Ratio and Gastrointestinal Bleeding After Coronary Artery Bypass Grafting in the Sensitivity Analysis Excluding the MIMIC-IV Cohort	29

Figure S8. Relationship Between the Stress Hyperglycemia Ratio and In-Hospital Mortality in Patients Undergoing Coronary Artery Bypass Grafting in the Sensitivity Analysis Excluding the MIMIC-IV Cohort	30

Figure S9. Associations of the Stress Hyperglycemia Ratio With Clinical Outcomes After Adjustment for Study Center in Restricted Cubic Spline Models	31

Figure S10. Association Between the Stress Hyperglycemia Ratio and Overt Gastrointestinal Bleeding After Coronary Artery Bypass Grafting	32

Figure S11. ROC Curve Analysis of SHR and Other Indicators in Predicting GIBCG	33

Figure S12. ROC Curve Analysis of SHR and Other Indicators in Predicting In-Hospital Mortality	34

Supplementary Methods.

**Detailed description of multiple imputation techniques**

To address missing data in our dataset, we employed the Multivariate Imputation by Chained Equations (MICE) algorithm using R software (version 4.2.3). This method, widely adopted in epidemiology and the social sciences, relies on the principle of Fully Conditional Specification (FCS). Each variable with missing values is imputed using a specific regression model, conditioned on the other variables in the dataset. The procedure is iterative and converges to a stable set of imputed values through successive updating.

The imputation process involved generating five complete datasets, which is the default and widely accepted number for multiple imputation in moderately sized datasets. A fixed random seed was specified to ensure reproducibility of the results. The type of imputation method used for each variable was automatically determined based on its data type: continuous variables were imputed using predictive mean matching, while categorical variables were imputed using logistic regression for binary outcomes or polytomous regression for nominal outcomes with more than two categories.

Following imputation, each of the five datasets was analyzed independently using logistic regression models, in which the binary outcome variable was regressed on all explanatory variables. The resulting estimates from the separate models were then pooled using Rubin’s rules, which provide combined estimates of coefficients and standard errors while accounting for the variability between imputations.All five imputed datasets were combined according to Rubin’s rules for downstream analyses and data export, rather than selecting a single dataset.

**References:**

1. Lee KJ, Carlin JB. Multiple imputation for missing data: fully conditional specification versus multivariate normal imputation. Am J Epidemiol. 2010 Mar 1;171(5):624–32. https://doi.org/10.1093/aje/kwp425

2. Austin PC, White IR, Lee DS, et al. Missing data in clinical research: a tutorial on multiple imputation. Can J Cardiol. 2021 Sep;37(9):1322–31.

**Table S1. The definition of each variable collected in this study**

| **Variables (n = 35)** | **Definition** |
| --- | --- |
| **Demographics** |  |
| Age | Age in years |
| Sex | Male or female |
| Body mass index | Weight in kg / height in m^2^ |
| Gastrointestinal bleeding history | Individual has previously experienced one or more episodes of gastrointestinal bleeding. |
| After percutaneous coronary intervention | Individual has previously undergone one or more percutaneous coronary interventions. |
| **Comorbidities** | **Documented in the medical history of patients or meets the following diagnostic criteria** |
| Anemia | Hemoglobin concentration below 130 g/L for men and below 120 g/L for women, except in acute anemia. |
| Coagulation disorders | Coagulation disorders caused by deficiencies or functional abnormalities of clotting factors, including hereditary conditions (such as hemophilia) and acquired cases, excluding coagulation dysfunctions that are caused by severe liver disease and those that occur acutely. |
| Hypertension | Recorded blood pressure >130/80 mmHg on three or more occasions. |
| Atrial fibrillation | Electrocardiogram showing no significant P waves and unequal R-R intervals owing to irregular ventricular responses. |
| Diabetes | Receiving anti-hyperglycemic medications including insulin. |
| Heart failure | Relying on information on history, physical examination, laboratory tests, cardiac imaging, and functional tests can confirm the diagnosis of a patient’s heart failure. |
| Cerebral vascular disease | A spectrum of diseases caused by cerebrovascular abnormalities primarily includes two major categories: ischemic (cerebral thrombosis and cerebral embolism) and hemorrhagic (cerebral hemorrhage). |
| Peripheral vascular disease | Ischemic diseases of the limbs; common types include peripheral arterial disease, varicose veins, and deep vein thrombosis. |
| Chronic kidney disease | Impairment of kidney structure and function (with a history of more than 3 months) |
| Gastrointestinal ulcer | Peptic ulcers occurring in the stomach and duodenum. |

| **Variables (n = 35)** | **Definition** |
| --- | --- |
| **Comorbidities** | **Documented in history or meets the following diagnostic criteria.** |
| Gastritis | Various chronic inflammatory lesions of the gastric mucosa typically include chronic superficial gastritis, chronic erosive gastritis, chronic atrophic gastritis, etc. |
| Hyperlipidemia | Hypercholesterolemia (total cholesterol, TC ≥ 5.2 mmol/L), hypertriglyceridemia (triglycerides, TG ≥ 1.7 mmol/L), hyper-low-density lipoprotein cholesterolemia (low-density lipoprotein cholesterol, LDL-C ≥ 3.4 mmol/L), and low high-density lipoprotein cholesterolemia (high |
|  | LDL cholesterol, HDL-C <1.0 mmol/L) |
| Valvular disease | Diseases in which the four valves in the heart, i.e., the mitral, aortic, tricuspid, and pulmonary valves, develop functional or structural abnormalities. Valvular disease may result in valve stenosis or valve closure insufficiency. |
| **Admission examination** |  |
| Stress Hyperglycemia Ratio | Stress Hyperglycemia Ratio (SHR) calculated based on blood glucose (ABG) and glycated hemoglobin (HbA1c). The formula is as follows: |
|  | SHR = ABG (mg/dl) / (28.7 * HbA1c (%) - 46.7). |
| White blood cell count, red blood cell count, platelets, hemoglobin, and hematocrit | Results from the patient’s first complete blood count after admission to the hospital. |
| Alanine aminotransferase, aspartate aminotransferase, bilirubin total, albumin, urea nitrogen, creatinine, and lactate dehydrogenase | Results from the patient’s first blood chemistry analysis after admission to the hospital. |
| Prothrombin time, activated partial thromboplastin time, and international normalized ratio | Results from the patient’s first coagulation test after admission to the hospital. |
| Ejection fraction | The ejection fraction from the patient’s first echocardiography after admission. |
| **Antithrombotic medications** |  |
| Single antiplatelet therapy | Previously, a single antiplatelet agent—such as aspirin, clopidogrel, ticagrelor, prasugrel, tirofiban, or eptifibatide—had been used. |
| Dual antiplatelet therapy | Combined use of aspirin and a P2Y12 receptor inhibitor—such as clopidogrel, ticagrelor, or prasugrel |
| Oral anticoagulants | Use of oral anticoagulants such as warfarin, rivaroxaban, apixaban, edoxaban, or dabigatran before surgery. |
| **Surgical characteristics** |  |
| Surgical duration | Surgical duration refers to the total time from the initial incision to the completion of the surgical procedure. |
| No. of grafts | No. of grafts refers to the number of blood vessel grafts used to bypass blocked coronary arteries during CABG surgery. |
| On pump | On pump refers to whether a heart-lung machine was used to circulate blood during CABG surgery. |
| On pump time | On pump time refers to the duration the patient is supported by the heart-lung machine during surgery. |
| Aortic occlusion time | Aortic occlusion time refers to the duration the aorta is clamped to stop blood flow during cardiac surgery. |

**Table S2. ICD-9/ICD-10 Codes are Used to Identify Chronic Comorbidities and Procedures**

| **Disease** | **ICD version** | **ICD code** |
| --- | --- | --- |
| Procedure：Coronary artery bypass grafting | 9 | '3610', '3611', '3612', '3613', '3614', '3615', '3616', '3617', '3619' |
|  | 10 | 'ICD codes that start with '021' and have long titles that include 'coronary artery' |
| Gastrointestinal bleeding | 9 | '53100', '53101', '53120', '53121', '53200', '53201', '53220', '53221', '53300', '53301', '53320', '53321', '53400', '53401', '53420', '53421', '53501', '53511', '53521', '53531', '53541', '53551', '53561', '53571', '5780', '5781', '5789' |
|  | 10 | 'K250', 'K252', 'K260', 'K262', 'K270', 'K272', 'K280', 'K282', 'K2901', 'K2961', 'K2971', 'K2981', 'K2991', 'K920', 'K921', 'K922' |
| Anemia | 9 | '2800','2801','2808','2809','2810','2811','2812','2813','2814','2818','2819','2820','2821','2822','2823','28240','28241','28242','28243','28244','28245','28246','28247','28249','2825','28260','28261','28262','28263','28264','28268','28269','2827','2828','2829','2830','28310','28311','28319','2832','2839','28401','28409','2841','28411','28412','28419','2842','28481','28489','2849','2850','2851','28521','28522','28529','2853','2858','2859' |
|  | 10 | 'D50','D500','D501','D508','D509','D51','D510','D511','D512','D513','D518','D519','D52','D520','D521','D528','D529','D53','D530','D531','D532','D538','D539','D55','E75242','D550','D551','D552','D553','D558','D559','D56','D560','D561','D562','D563','D564','D565','D568','D569','D57','D570','D5700','D5701','D5702','D571','D572','D5720','D5721','D57211','D57212','D57219','D573','D574','D5740','D5741','D57411','D57412','D57419','D578','D5780','D5781','D57811','D57812','D57819','D58','D580','D581','D582','D588','D589','D59','D590','D591','D592','D593','D594','D595','D596','D598','D599','D61','D610','D6101','D6109','D611','D612','D613','D618','D6181','D61810','D61811','D61818','D6182','D6189','D619','D61','D610','D6101','D6109','D611','D612','D613','D618','D6181','D61810','D61811','D61818','D6182','D6189','D619','D62','D63','D630','D631','D638','D64','D640','D641','D642','D643','D644','D648','D6481','D6489','D649' |
| Coagulation disorders | 9 | '2860','286','2862','2863','2864','2865','28652','28653','28659','2866','2867','2869' |
|  | 10 | 'D65','D66','D67','D68','D680','D681','D682','D683','D6831','D68311','D68312','D68318','D6832','D684','D685','D6851','D6852','D6859','D686','D6861','D6862','D6869','D688','D689','D69','D690','D691','D692','D693','D694','D6941','D6942','D6949','D695','D6951','D6959','D696','D698','D699' |

| **Disease** | **ICD version** | **ICD code** |
| --- | --- | --- |
| Hypertension | 9 | '4010','4011','4019' |
|  | 10 | 'I10' |
| Atrial fibrillation | 9 | '42731' |
|  | 10 | 'I48','I480','I481','I4811','I4819','I482','I4820','I4821','I483','I484','I489','I4891','I4892' |
| Diabetes | 9 | '2500','2501','2502','2503','2508','2509' |
|  | 10 | 'E100','E10l','E106','E108','E109','E110','E111','E116','E118','E119','E120','E121','E126','E128','E129','E130','E131','E136','E138','E139','E140', 'E141','E146','E148','E149' |
| Heart failure | 9 | '4281','42820','42821','42822','42823','42830','42831','42832','42833','42840','42841','42842','42843','4289','39891','40201','40211','40291','40401','40403','40411','40413','40491','40493','4254','4255','4257','4258','4259' |
|  | 10 | 'I43','I50','I099','I110','I130','I132','I255','I42','I426','I427','I428','I429','P290',' |
| Cerebral vascular disease | 9 | '430','431','4320','4321','4329','43300','43301','43310','43311','43320','43321','43330','43331','43380','43381','36234' |
|  | 10 | 'G45 to G459', 'G46 to G468', 'I60 to I609', 'I61 to I619', 'I62 to I629', 'I63 to I639', 'I65 to I659', 'I66 to I669', 'I67 to I679', 'I68 to I688', 'I69 to I699', 'H340 to H3403' |
| Peripheral vascular disease | 9 | '4400','4401','44020','44021','44022','44023','44024','44029','44030','44031','44032','4404','4408','4409','44100','44101','44102','44103','4411','4412','4413','4414','4415','4416','4417','4419','0930','4431','44321','44322','44323','44324','44329','44381','44382','44389','4439' |
|  | 10 | 'I70 to I7075', 'I7076 to I709', 'I71', 'I710 to I719', 'I731', 'I738', 'I7381', 'I7389', 'I739', 'I771', 'I790', 'K551', 'K558', 'K559', 'Z958', 'Z9581', 'Z95810 to Z95812', 'Z95818', 'Z9582', 'Z95820', 'Z95828', 'Z959' |
| Chronic kidney disease | 9 | '5820','5821','5822','5824','58281','58289','5829','5851','5852','5853','5854','5855','5856','5859','586','V560','V561','V562','V5631','V5632','V568','5830','5831','5832','5834','5836','5837','40301','40311','40391','40402','40403','40412','40413','40492','40493' |
|  | 10 | 'N18','N181','N182','N183','N184','N185','N186','N189','N19','I120','I131','I1310','I1311','N032','N033','N034','N035','N036','N037','N052','N053','N054','N055','N056','N057','N250','Z490','Z4901','Z4902','Z940','Z992' |
| Gastrointestinal ulcer | 9 | '53140','53141','53150','53151','53160','53161','53520','53170','53171','53240','53241','53250','53251','53260','53261','53270','5'3271','53340','53341','53350','53351','53360','53361','53370','53371','53440','53441','53450','53451','53460','53461','53470','53471' |
|  | 10 | 'K25','K254','K255','K256','K257','K26','K264','K265','K266','K267','K27','K274','K275','K276','K277','K28','L02225','K284','K285','K286','K287' |

| **Disease** | **ICD version** | **ICD code** |
| --- | --- | --- |
| Gastritis（Non acute） | 9 | '53501','53510','53511','53521','53530','53531','53540','53541','53550','53551','53560','53561','53570','53571' |
|  | 10 | 'K292','K2920','K2921','K293','K2930','K2931','K294','K2940','K2941','K295','K2950','K2951','K296','K2960','K2961','K297','K2970','K2971','K298','K2980','K2981','K299','K2990','K2991' |
| Hyperlipidemia | 9 | '2720','2721','2722','2723','2724' |
|  | 10 | 'E78','E780','E7800','E7801','E781','E782','E783','E784','E7841','E7849','E785' |
| Valvular disease | 9 | '3940','3941','3942','3949','3950','3951','3952','3959','3960','3961','3962','3963','3968','3969','3970','3971','3979','4240','4241','4242','4243' |
|  | 10 | 'I34','I340','I341','I342','I348','I349','I35','I350','I351','I352','I358','I359','I36','I360','I361','I362','I368','I369','I37','I370','I371','I372','I378','I379','I05','I050','I051','I052','I058','I059','I06','I060','I061','I062','I068','I069','I07','I070','I071','I072','I078','I079','I08','I080','I081','I082','I083','I088','I089' |

* The ICD_code for certain diseases is abbreviated as it has too many codes and takes up too much space.

Abbreviation: ICD-9, international classification of diseases, 9th revision; ICD-10, international classification of diseases, 10th revision.

**Table S3. Confounding factors adjusted in the multivariate logistic regression models**

| Logistic regression | Adjusted variables |
| --- | --- |
| The outcome was GIBCG | age, sex, body mass index, gastrointestinal bleeding history, anemia, coagulation disorders, hypertension, atrial fibrillation, diabetes, heart failure, cerebral vascular disease, peripheral vascular disease, chronic renal insufficiency, gastrointestinal ulcer, gastritis, hyperlipidemia, after percutaneous coronary intervention, valvular disease, white blood cell count, red blood cell count, platelet count, hemoglobin, hematocrit, alanine aminotransferase, aspartate aminotransferase, total bilirubin, albumin, urea, creatinine, prothrombin time, activated partial thromboplastin time, international normalized ratio, ejection fraction, lactate dehydrogenase, single antiplatelet therapy, dual antiplatelet therapy, oral anticoagulants, surgical duration, number of grafts, on pump, on pump time,and aortic occlusion time. |
| The outcome was in-hospital mortality | age, sex, body mass index, anemia, coagulation disorders, hypertension, atrial fibrillation, diabetes, heart failure, cerebral vascular disease, chronic renal insufficiency, after percutaneous coronary intervention, valvular disease, white blood cell count, red blood cell count, platelet count, hemoglobin, hematocrit, alanine aminotransferase, aspartate aminotransferase, total bilirubin, albumin, creatinine, international normalized ratio, ejection fraction, lactate dehydrogenase, single antiplatelet therapy, dual antiplatelet therapy, oral anticoagulants, surgical duration, number of grafts, on pump, on pump time,and aortic occlusion time. |
| Subgroup analysis of in-hospital mortality: core covariates for adjustment | age, sex, heart failure, chronic renal insufficiency, hemoglobin, international normalized ratio, ejection fraction, white blood cell count |

* In the primary adjusted model for in-hospital mortality, 34 pre-specified covariates were included to control for potential confounding as much as possible. Given the limited number of events in some subgroups, a simplified adjustment model with pre-specified key clinical covariates related to mortality risk was used in the subgroup analyses. This approach aimed to reduce the risk of overfitting, improve the stability and robustness of the effect estimates, and still adequately adjust for major confounders.

**Table S4. Description of the Data Types and Missing Values for Each Variable**

| Variables | Data Types | Missing Values |
| --- | --- | --- |
| **Demographics** |  |  |
| Age | Continuous | 15 (0.09%) |
| Sex | Categorical | 68 (0.41%) |
| Body mass index | Continuous | 533 (3.24%) |
| Gastrointestinal bleeding history | Categorical | 0 (0%) |
| After percutaneous coronary intervention | Categorical | 0 (0%) |
| **Chronic comorbidities** |  |  |
| Anemia | Categorical | 0 (0%) |
| Coagulation disorders | Categorical | 0 (0%) |
| Hypertension | Categorical | 0 (0%) |
| Atrial fibrillation | Categorical | 0 (0%) |
| Diabetes | Categorical | 0 (0%) |
| Heart failure | Categorical | 0 (0%) |
| Cerebral vascular disease | Categorical | 0 (0%) |
| Peripheral vascular disease | Categorical | 0 (0%) |
| Chronic kidney disease | Categorical | 0 (0%) |
| Gastrointestinal ulcer | Categorical | 0 (0%) |
| Gastritis | Categorical | 0 (0%) |
| Hyperlipidemia | Categorical | 0 (0%) |
| Valvular disease | Categorical | 0 (0%) |
| **Admission examination** |  |  |
| Stress Hyperglycemia Ratio | Continuous | 97 (0.59%) |
| White blood cells | Continuous | 52 (0.32%) |
| Red blood cells | Continuous | 53 (0.32%) |
| Platelets | Continuous | 55 (0.33%) |
| Hemoglobin | Continuous | 75 (0.46%) |
| Hematocrit | Continuous | 53 (0.32%) |
| Alanine aminotransferase | Continuous | 128 (0.78%) |
| Aspartate aminotransferase | Continuous | 98 (0.6%) |
| Total bilirubin | Continuous | 744 (4.53%) |
| Albumin | Continuous | 765 (4.65%) |
| Urea | Continuous | 219 (1.33%) |
| Creatinine | Continuous | 60 (0.36%) |
| Prothrombin time | Continuous | 99 (0.6%) |
| Activated partial thromboplastin time | Continuous | 106 (0.64%) |
| International Normalized Ratio | Continuous | 102 (0.62%) |
| Lactate dehydrogenase | Continuous | 433 (2.63%) |
| Ejection fraction | Continuous | 1135 (6.9%) |
| **Antithrombotic medications** |  |  |
| Single antiplatelet therapy | Categorical | 0 (0%) |
| Dual antiplatelet therapy | Categorical | 0 (0%) |
| Oral anticoagulants | Categorical | 0 (0%) |
| **Surgical characteristics** |  |  |
| Surgical duration | Continuous | 0 (0%) |
| No. of grafts | Ordinal Categorical | 0 (0%) |
| On pump | Categorical | 0 (0%) |
| On pump time | Continuous | 89 (0.54%) |
| Aortic occlusion time | Continuous | 89 (0.54%) |

**Table S5. Comparison of Sample Characteristics Before and After Imputation**

| Characteristic | Before_interpolation | After_interpolation | p value |
| --- | --- | --- | --- |
| Demographics |  |  |  |
| Age, median (IQR), y | 64 (13) | 64 (13) | 0.981 |
| Sex, n (%) |  |  | 0.964 |
| Male | 12180 (74.4%) | 12227 (74.37%) |  |
| Female | 4192 (25.6%) | 4213 (25.63%) |  |
| Body mass index, median (IQR) | 25.4 (4.26) | 25.4 (4.24) | 0.906 |
| Admission examination |  |  |  |
| Stress Hyperglycemia Ratio, median (IQR) | 0.792 (0.199) | 0.792 (0.199) | 0.982 |
| White blood cell count, median (IQR) , ×10^9/L | 6.8 (2.84) | 6.8 (2.84) | 0.988 |
| Red blood cell count, median (IQR), ×10^12/L | 4.39 (0.77) | 4.39 (0.77) | 0.987 |
| Platelet count, median (IQR), ×10^9/L | 204 (79) | 204 (79) | 0.952 |
| Hemoglobin, median (IQR), g/L | 135 (25) | 135 (25) | 0.985 |
| Hematocrit, median (IQR), Proportion of 1.0 | 0.395 (0.068) | 0.395 (0.068) | 0.988 |
| Alanine aminotransferase, median (IQR), µkat/L | 0.351 (0.301) | 0.351 (0.301) | 1.000 |
| Aspartate aminotransferase, median (IQR), µkat/L | 0.334 (0.184) | 0.334 (0.184) | 0.969 |
| Bilirubin total, median (IQR), µmol/L | 11 (6.52) | 11 (6.56) | 0.915 |
| Albumin, median (IQR), g/L | 42 (5.1) | 42 (5.1) | 0.951 |
| Urea, median (IQR), mmol/L | 5.84 (2.39) | 5.84 (2.39) | 0.959 |
| Creatinine, median (IQR), μmol/L | 74.7 (22.8) | 74.7 (22.8) | 0.961 |
| Prothrombin time, median (IQR), s | 11.4 (1.1) | 11.4 (1.1) | 0.939 |
| Activated partial thromboplastin time, median (IQR), s | 30.6 (4.7) | 30.6 (4.7) | 0.996 |
| International normalized ratio, median (IQR) | 1.01 (0.09) | 1.01 (0.09) | 0.991 |
| Lactate dehydrogenase, median (IQR), µkat/L | 2.99 (0.87) | 2.99 (0.87) | 0.890 |
| Ejection fraction, No. (%) |  |  | 0.998 |
| ≥55% | 11443 (74.77%) | 12292 (74.77%) |  |
| 45-55% | 2266 (14.81%) | 2427 (14.76%) |  |
| 30-45% | 1470 (9.6%) | 1583 (9.63%) |  |
| ＜30% | 126 (0.82%) | 138 (0.84%) |  |
| Surgical characteristics |  |  |  |
| On pump time, median (IQR), min | 159 (60) | 159 (64) | 0.845 |
| Aortic occlusion time, median (IQR), min | 91 (30) | 91 (30) | 0.847 |

**Table S6. Data Volume and Baseline Characteristics of Each Center**

| Characteristic | Anzhen | Chaoyang | Drum Tower | Luhe | MIMIC |
| --- | --- | --- | --- | --- | --- |
|  | n=12194 | n=904 | n=2430 | n=616 | n=296 |
| Demographics |  |  |  |  |  |
| Age, median (IQR), y | 63 (12) | 63 (12) | 64 (14) | 67 (13) | 73.64 (14.68) |
| Sex, n (%) |  |  |  |  |  |
| Male | 9206 (75.5%) | 690 (76.33%) | 1707 (70.25%) | 426 (69.16%) | 198 (66.89%) |
| Female | 2988 (24.5%) | 214 (23.67%) | 723 (29.75%) | 190 (30.84%) | 98 (33.11%) |
| Body mass index, median (IQR) | 25.59 (4.07) | 25.6 (4.28) | 24.4 (4.2) | 25.01 (4.47) | 30.09 (7.31) |
| Gastrointestinal bleeding history, n (%) | 92 (0.75%) | 8 (0.88%) | 21 (0.86%) | 10 (1.62%) | 30 (10.14%) |
| After percutaneous coronary intervention, n (%) | 1426 (11.69%) | 93 (10.29%) | 232 (9.55%) | 61 (9.9%) | 28 (9.46%) |
| Chronic comorbidities |  |  |  |  |  |
| Anemia, n (%) | 1433 (11.75%) | 90 (9.96%) | 254 (10.45%) | 73 (11.85%) | 122 (41.22%) |
| Coagulation disorders, n (%) | 216 (1.77%) | 18 (1.99%) | 46 (1.89%) | 11 (1.79%) | 45 (15.2%) |
| Hypertension, n (%) | 5631 (46.18%) | 511 (56.53%) | 1449 (59.63%) | 338 (54.87%) | 131 (44.26%) |
| Atrial fibrillation, n (%) | 765 (6.27%) | 62 (6.86%) | 154 (6.34%) | 40 (6.49%) | 85 (28.72%) |
| Diabetes, n (%) | 4605 (37.76%) | 371 (41.04%) | 933 (38.40%) | 237 (38.47%) | 100 (33.78%) |
| Heart failure, n (%) | 4619 (37.88%) | 345 (38.16%) | 983 (40.45%) | 281 (45.62%) | 146 (49.32%) |
| Cerebral vascular disease, n (%) | 1836 (15.06%) | 137 (15.15%) | 431 (17.74%) | 125 (20.29%) | 53 (17.91%) |
| Peripheral vascular disease, n (%) | 419 (3.44%) | 26 (2.88%) | 77 (3.17%) | 26 (4.22%) | 21 (7.09%) |
| Chronic kidney disease, n (%) | 567 (4.65%) | 38 (4.2%) | 115 (4.73%) | 27 (4.38%) | 113 (38.18%) |
| Gastrointestinal ulcer, n (%) | 183 (1.5%) | 14 (1.55%) | 39 (1.6%) | 10 (1.62%) | 11 (3.72%) |
| Gastritis, n (%) | 168 (1.38%) | 20 (2.21%) | 35 (1.44%) | 17 (2.76%) | 10 (3.38%) |
| Hyperlipidemia, n (%) | 7695 (63.1%) | 541 (59.84%) | 1498 (61.65%) | 368 (59.74%) | 201 (67.91%) |
| Valvular disease, n (%) | 2241 (18.38%) | 148 (16.37%) | 500 (20.58%) | 129 (20.94%) | 107 (36.15%) |
| Admission examination |  |  |  |  |  |
| Stress Hyperglycemia Ratio, median (IQR) | 0.8 (0.23) | 0.79 (0.23) | 0.79 (0.12) | 0.79 (0.13) | 0.97 (0.35) |
| White blood cell count, median (IQR) , ×10^9/L | 6.92 (2.85) | 6.62 (2.48) | 6.3 (2.4) | 6.27 (2.48) | 11.35 (7.67) |
| Red blood cell count, median (IQR), ×10^12/L | 4.41 (0.77) | 4.43 (0.73) | 4.38 (0.72) | 4.35 (0.71) | 3.25 (0.79) |
| Platelet count, median (IQR), ×10^9/L | 207 (79) | 208 (77) | 198 (73) | 197.5 (82) | 176.5 (136) |
| Hemoglobin, median (IQR), g/L | 136 (26) | 138 (24) | 134 (22) | 134 (21) | 98 (23) |
| Hematocrit, median (IQR), Proportion of 1.0 | 0.395 (0.069) | 0.401 (0.064) | 0.398 (0.06) | 0.394 (0.067) | 0.295 (0.063) |
| Alanine aminotransferase, median (IQR), µkat/L | 0.351 (0.301) | 0.334 (0.267) | 0.361 (0.321) | 0.317 (0.267) | 0.401 (0.564) |
| Aspartate aminotransferase, median (IQR), µkat/L | 0.317 (0.167) | 0.317 (0.134) | 0.371 (0.239) | 0.334 (0.187) | 0.618 (0.99) |
| Bilirubin total, median (IQR), µmol/L | 11.04 (6.42) | 10.68 (6.59) | 10.8 (6.73) | 11 (6.8) | 10.26 (13.25) |
| Albumin, median (IQR), g/L | 42.7 (4.8) | 42.85 (4.77) | 39.6 (4) | 40.5 (4.7) | 33 (8) |
| Urea, median (IQR), mmol/L | 5.76 (2.27) | 5.99 (2.25) | 6.1 (2.6) | 5.87 (2.4) | 9.64 (8.93) |
| Creatinine, median (IQR), μmol/L | 74.85 (21.6) | 76.25 (20.88) | 71 (24.7) | 74.25 (27) | 106.08 (79.56) |
| Prothrombin time, median (IQR), s | 11.4 (1.1) | 11.2 (1.1) | 11.5 (1.3) | 11.4 (1.3) | 14.4 (4.6) |
| Activated partial thromboplastin time, median (IQR), s | 31 (4.4) | 31 (4.1) | 27.8 (4.1) | 29.3 (5.2) | 31.8 (12.8) |
| International normalized ratio, median (IQR) | 1.01 (0.09) | 1 (0.08) | 1.01 (0.12) | 1.02 (0.11) | 1.3 (0.5) |
| Lactate dehydrogenase, median (IQR), µkat/L | 2.939 (0.768) | 2.906 (0.718) | 3.023 (1.136) | 3.039 (1.152) | 5.06 (3.023) |
| Ejection fraction, No. (%) |  |  |  |  |  |
| ≥55% | 9466 (77.63%) | 685 (75.77%) | 1593 (65.55%) | 439 (71.27%) | 109 (36.82%) |
| 45-55% | 1827 (14.98%) | 115 (12.72%) | 317 (13.05%) | 92 (14.94%) | 76 (25.68%) |
| 30-45% | 1019 (8.36%) | 89 (9.84%) | 324 (13.33%) | 79 (12.82%) | 72 (24.32%) |
| ＜30% | 57 (0.47%) | 10 (1.11%) | 26 (1.07%) | 6 (0.97%) | 39 (13.18%) |
| Antithrombotic medications |  |  |  |  |  |
| Single antiplatelet therapy, n (%) | 2004 (16.43%) | 156 (17.26%) | 458 (18.85%) | 117 (18.99%) | 127 (42.91%) |
| Dual antiplatelet therapy, n (%) | 2678 (21.96%) | 201 (22.23%) | 604 (24.86%) | 144 (23.38%) | 44 (14.86%) |
| Oral anticoagulants, n (%) | 99 (0.81%) | 10 (1.11%) | 28 (1.15%) | 7 (1.14%) | 9 (3.04%) |
| Surgical characteristics |  |  |  |  |  |
| Surgical duration, median (IQR), h | 4.12 (1.33) | 4.13 (1.12) | 5.5 (1.87) | 5 (2) | 4.75 (1.73) |
| No. of grafts, median (IQR) | 3 (1) | 3 (1) | 3 (2) | 2 (1) | 3 (2) |
| On pump, n (%) | 6106 (50.07%) | 448 (49.56%) | 1240 (51.03%) | 316 (51.3%) | 140 (47.30%) |
| On pump time, median (IQR), min | 176 (69) | 200 (95) | 177 (91) | 162 (83) | 203 (100) |
| Aortic occlusion time, median (IQR), min | 125 (63) | 142.5 (77.5) | 129 (75) | 112 (64) | 146 (91.8) |
| Incidence of GIBCG, n (%) | 740 (6.07%) | 58 (6.41%) | 146 (6.01%) | 38 (6.17%) | 176 (59.46%) |
| In-hospital mortality, n (%) | 135 (1.11%) | 10 (1.11%) | 42 (1.73%) | 7 (1.14%) | 20 (6.76%) |

**Table S7. Comparison of the SHR Sampling-to-Surgery Interval Between Patients With and Without Postoperative Gastrointestinal Bleeding**

| Characteristic | Overall, N = 16440 | No GIB, N = 15282 | GIB, N = 1158 | P value |
| --- | --- | --- | --- | --- |
| Sampling-to-surgery interval, median (IQR), d | 0.876 (0.239) | 0.875 (0.239) | 0.878 (0.246) | 0.06 |

**Table S8. Number of In-Hospital Deaths Across All Subgroups**

| Subgroups | Deaths |
| --- | --- |
| Age＞=65 (n=8008) | 152 |
| Age＜65 (n=8432) | 62 |
| Female (n=4213) | 67 |
| Male (n=12227) | 147 |
| Diabetes_Ⅱ_Yes (n=6246) | 88 |
| Diabetes_Ⅱ_No (n=10194) | 126 |
| Hypertension_Yes (n=8060) | 123 |
| Hypertension_No (n=8380) | 91 |
| Heart_Failure_Yes (n=6374) | 132 |
| Heart_Failure_No (n=10066) | 82 |

**Table S9 Demographic and Baseline Characteristics of Patients Stratified by Year of Surgery**

| Characteristic | 2018 | 2019 | 2020 | 2021 | 2022 | 2023 | 2024 | p value |
| --- | --- | --- | --- | --- | --- | --- | --- | --- |
|  | n=1487 | n=1710 | n=1721 | n=3234 | n=3320 | n=3605 | n=1067 |  |
| Demographics |  |  |  |  |  |  |  |  |
| Age, median (IQR), y | 64 (13) | 64 (12) | 65 (12) | 64 (13) | 64 (13) | 64 (13) | 64 (12) | 0.242 |
| Sex, n (%) |  |  |  |  |  |  |  | 0.958 |
| Male | 1113 (74.85%) | 1267 (74.09%) | 1285 (74.67%) | 2410 (74.52%) | 2463 (74.19%) | 2706 (75.06%) | 785 (73.57%) |  |
| Female | 374 (25.15%) | 443 (25.91%) | 436 (25.33%) | 824 (25.48%) | 857 (25.81%) | 899 (24.94%) | 282 (26.43%) |  |
| Gastrointestinal bleeding history, n (%) | 11 (0.74%) | 20 (1.17%) | 15 (0.87%) | 22 (0.68%) | 29 (0.87%) | 20 (0.55%) | 14 (1.31%) | 0.124 |
| After percutaneous coronary intervention, n (%) | 149 (10.02%) | 213 (12.46%) | 192 (11.16%) | 360 (11.13%) | 407 (12.26%) | 408 (11.32%) | 111 (10.4%) | 0.202 |
| Chronic comorbidities |  |  |  |  |  |  |  |  |
| Anemia, n (%) | 172 (11.57%) | 200 (11.7%) | 191 (11.1%) | 319 (9.86%) | 400 (12.05%) | 412 (11.43%) | 126 (11.81%) | 0.158 |
| Coagulation disorders, n (%) | 18 (1.21%) | 18 (1.05%) | 15 (0.87%) | 33 (1.02%) | 30 (0.9%) | 30 (0.83%) | 20 (1.87%) | 0.109 |
| Hypertension, n (%) | 729 (49.02%) | 798 (46.67%) | 848 (49.27%) | 1564 (48.36%) | 1650 (49.7%) | 1798 (49.88%) | 542 (50.8%) | 0.289 |
| Diabetes, n (%) | 542 (36.45%) | 689 (40.29%) | 662 (38.47%) | 1236 (38.22%) | 1269 (38.22%) | 1335 (37.03%) | 413 (38.71%) | 0.299 |
| Heart failure, n (%) | 556 (37.39%) | 659 (38.54%) | 672 (39.05%) | 1222 (37.79%) | 1278 (38.49%) | 1409 (39.08%) | 432 (40.49%) | 0.684 |
| Cerebral vascular disease, n (%) | 227 (15.27%) | 297 (17.37%) | 260 (15.11%) | 486 (15.03%) | 506 (15.24%) | 589 (16.34%) | 164 (15.37%) | 0.312 |
| Chronic kidney disease, n (%) | 68 (4.57%) | 85 (4.97%) | 94 (5.46%) | 136 (4.21%) | 126 (3.8%) | 194 (5.38%) | 44 (4.12%) | 0.021 |
| Admission examination |  |  |  |  |  |  |  |  |
| Stress Hyperglycemia Ratio, median (IQR) | 0.79 (0.18) | 0.8 (0.2) | 0.79 (0.2) | 0.79 (0.19) | 0.79 (0.2) | 0.8 (0.19) | 0.8 (0.21) | 0.214 |
| White blood cell count, median (IQR) , ×10^9/L | 6.72 (2.94) | 6.775 (2.79) | 6.8 (2.68) | 6.72 (2.74) | 6.81 (2.82) | 6.77 (2.76) | 6.82 (3) | 0.826 |
| Red blood cell count, median (IQR), ×10^12/L | 4.41 (0.77) | 4.4 (0.78) | 4.4 (0.77) | 4.41 (0.77) | 4.4 (0.78) | 4.39 (0.73) | 4.41 (0.78) | 0.879 |
| Platelet count, median (IQR), ×10^9/L | 204 (80) | 206 (79.3) | 204 (80) | 204 (77) | 204 (79) | 206 (78) | 204 (79) | 0.686 |
| Hemoglobin, median (IQR), g/L | 136 (24) | 136 (26) | 136 (25) | 136 (24.3) | 136 (24) | 136 (24) | 136 (25) | 0.912 |
| Albumin, median (IQR), g/L | 42.1 (5.1) | 42 (5) | 42.1 (4.9) | 42.2 (4.9) | 42.3 (5) | 42.1 (5) | 41.9 (4.8) | 0.285 |
| Creatinine, median (IQR), μmol/L | 74 (23) | 74.7 (23) | 74.2 (22.6) | 74.2 (21.7) | 75.1 (21.6) | 74.5 (22.1) | 73.8 (23.6) | 0.716 |
| International normalized ratio, median (IQR) | 1.01 (0.1) | 1.01 (0.1) | 1.01 (0.09) | 1.01 (0.1) | 1.01 (0.1) | 1.01 (0.09) | 1.01 (0.09) | 0.502 |
| Lactate dehydrogenase, median (IQR), µkat/L | 2.97 (0.89) | 2.97 (0.85) | 3.01 (0.85) | 2.99 (0.87) | 2.96 (0.82) | 2.99 (0.84) | 2.97 (0.8) | 0.303 |
| Ejection fraction, No. (%) |  |  |  |  |  |  |  | 0.526 |
| ≥55% | 1131 (76.06%) | 1299 (75.96%) | 1309 (76.06%) | 2428 (75.08%) | 2514 (75.72%) | 2706 (75.06%) | 796 (74.6%) |  |
| 45-55% | 218 (14.66%) | 249 (14.56%) | 254 (14.76%) | 474 (14.66%) | 482 (14.52%) | 531 (14.73%) | 143 (13.4%) |  |
| 30-45% | 125 (8.41%) | 156 (9.12%) | 150 (8.72%) | 318 (9.83%) | 302 (9.1%) | 340 (9.43%) | 120 (11.25%) |  |
| ＜30% | 13 (0.87%) | 6 (0.35%) | 8 (0.46%) | 14 (0.43%) | 22 (0.66%) | 28 (0.78%) | 8 (0.75%) |  |
| Surgical characteristics |  |  |  |  |  |  |  |  |
| Surgical duration, median (IQR), h | 4.17 (1.36) | 4.12 (1.37) | 4.29 (1.42) | 4.25 (1.33) | 4.29 (1.29) | 4.27 (1.42) | 4.16 (1.46) | 0.127 |
| No. of grafts, median (IQR) | 3 (1) | 3 (1) | 3 (1) | 3 (1) | 3 (1) | 3 (1) | 3 (1) | 0.345 |
| On pump, n (%) | 728 (48.96%) | 859 (50.23%) | 872 (50.67%) | 1629 (50.37%) | 1696 (51.08%) | 1822 (50.54%) | 534 (50.05%) | 0.919 |
| On pump time, median (IQR), min | 156 (82) | 160 (79) | 156 (84) | 158 (82) | 159.5 (86) | 160 (82) | 161 (84) | 0.573 |
| Aortic occlusion time, median (IQR), min | 89 (76.1) | 89 (77) | 90 (79) | 95 (78.2) | 91.5 (82) | 91 (77) | 90 (82.3) | 0.705 |
| Incidence of GIBCG, n (%) | 84 (5.65%) | 100 (5.85%) | 104 (6.04%) | 191 (5.91%) | 205 (6.17%) | 226 (6.27%) | 72 (6.75%) | 0.926 |
| In-hospital mortality, n (%) | 18 (1.21%) | 23 (1.35%) | 16 (0.93%) | 34 (1.05%) | 42 (1.27%) | 40 (1.11%) | 19 (1.78%) | 0.493 |

**Table S10. Associations Between Stress Hyperglycemia Ratio Quartiles and Clinical Outcomes After Adjustment for Study Center as a Fixed-Effect Covariate**

| SHR | GIBCG | |  | In-hospital Mortality | |
| --- | --- | --- | --- | --- | --- |
|  | OR (95%CI) | P Value |  | OR (95%CI) | P Value |
| Quartile 1 | 1.423 (1.135-1.788) | 0.002 |  | 1.992 (1.229-3.282) | 0.006 |
| Quartile 2 | Reference |  |  | Reference |  |
| Quartile 3 | 1.278 (1.018-1.607) | 0.035 |  | 1.622 (1.017-2.657) | 0.046 |
| Quartile 4 | 2.919 (2.375-3.605) | ＜0.001 |  | 2.138 (1.346-3.471) | 0.002 |

Table S11. The proportion of overt gastrointestinal bleeding events among all gastrointestinal bleeding events.

|  | Anzhen | Chaoyang | Drum Tower | Luhe | MIMIC |
| --- | --- | --- | --- | --- | --- |
|  | n=740 | n=58 | n=146 | n=38 | n=176 |
| Proportion of overt GIB (%) | 19.05% | 22.41% | 21.23% | 23.68% | 28.41% |

**Figure S1. Subgroup Analyses of the Association Between SHR and GIBCG in the Unadjusted Models.**

(A) Patients aged over 65; (B) patients aged under 65; (C) female subgroup; (D) male subgroup; (E) patients with diabetes; (F) patients without diabetes; (G) patients with hypertension; (H) patients without hypertension; (I) patients with heart failure; (J) patients without heart failure.

**Figure S2. Subgroup Analyses of the Association Between SHR and GIBCG in the Adjusted Models.**

(A) Patients aged over 65; (B) patients aged under 65; (C) female subgroup; (D) male subgroup; (E) patients with diabetes; (F) patients without diabetes; (G) patients with hypertension; (H) patients without hypertension; (I) patients with heart failure; (J) patients without heart failure.

**Figure S3. Subgroup Analyses of the Association Between SHR and In-Hospital Mortality in the Unadjusted Models.**

(A) Patients aged over 65; (B) patients aged under 65; (C) female subgroup; (D) male subgroup; (E) patients with diabetes; (F) patients without diabetes; (G) patients with hypertension; (H) patients without hypertension; (I) patients with heart failure; (J) patients without heart failure.

**Figure S4. Subgroup Analyses of the Association Between SHR and In-Hospital Mortality in the Adjusted Models.**

(A) Patients aged over 65; (B) patients aged under 65; (C) female subgroup; (D) male subgroup; (E) patients with diabetes; (F) patients without diabetes; (G) patients with hypertension; (H) patients without hypertension; (I) patients with heart failure; (J) patients without heart failure.

**Figure S5. Subgroup Analysis of the Association Between SHR and GIBCG in Patients Without a History of Gastrointestinal Bleeding**

(A) Comparison of the risk of GIBCG among patients with different SHR levels in the subgroup of patients without a history of gastrointestinal bleeding.

(B) Restricted cubic spline: A U-shaped relationship between SHR and GIBCG in the unadjusted model.

(C) Restricted cubic spline: The U-shaped relationship persisted in the adjusted model.

**Figure S6. Subgroup analyses of the association between SHR and In-Hospital Mortality in Patients Without a History of Gastrointestinal Bleeding**

(A) Comparison of in-hospital mortality risk among patients with different SHR levels in the subgroup of patients without a history of gastrointestinal bleeding.

(B) Restricted cubic spline: A U-shaped relationship between SHR and in-hospital mortality in the unadjusted model.

(C) Restricted cubic spline: The U-shaped relationship persisted in the adjusted model.

**Figure S7. Relationship Between the Stress Hyperglycemia Ratio and Gastrointestinal Bleeding After Coronary Artery Bypass Grafting in the Sensitivity Analysis Excluding the MIMIC-IV Cohort**

(A) Comparison of the risk of GIBCG among patients with different SHR levels in the sensitivity analysis excluding the MIMIC-IV cohort.

(B) Restricted cubic spline: A U-shaped relationship between SHR and in-hospital mortality in the unadjusted model.

(C) Restricted cubic spline: The U-shaped relationship persisted in the adjusted model.

**Figure S8. Relationship Between the Stress Hyperglycemia Ratio and In-Hospital Mortality in Patients Undergoing Coronary Artery Bypass Grafting in the Sensitivity Analysis Excluding the MIMIC-IV Cohort**

(A) Comparison of in-hospital mortality risk among patients with different SHR levels in the sensitivity analysis excluding the MIMIC-IV cohort.

(B) Restricted cubic spline: A U-shaped relationship between SHR and in-hospital mortality in the unadjusted model.

(C) Restricted cubic spline: The U-shaped relationship persisted in the adjusted model.

**Figure S9. Associations of the Stress Hyperglycemia Ratio With Clinical Outcomes After Adjustment for Study Center in Restricted Cubic Spline Models**

Restricted cubic spline curves showing the associations of the stress hyperglycemia ratio with clinical outcomes after adjustment for study center. (A) Gastrointestinal bleeding. (B) In-hospital mortality.

**Figure S10. Association Between the Stress Hyperglycemia Ratio and Overt Gastrointestinal Bleeding After Coronary Artery Bypass Grafting**

(A) Comparison of the risk of overt GIBCG among patients with different SHR levels

(B) Restricted cubic spline: A U-shaped relationship between SHR and in-hospital mortality in the unadjusted model.

(C) Restricted cubic spline: The U-shaped relationship persisted in the adjusted model.

**Figure S11. ROC Curve Analysis of SHR and Other Indicators in Predicting GIBCG**

**Figure S12. ROC Curve Analysis of SHR and Other Indicators in Predicting In-Hospital Mortality**
